# Analyzing and Modeling the Kinetics of Amyloid Beta Pores Associated with Alzheimer’s Disease Pathology

**DOI:** 10.1371/journal.pone.0137357

**Published:** 2015-09-08

**Authors:** Ghanim Ullah, Angelo Demuro, Ian Parker, John E. Pearson

**Affiliations:** 1 Department of Physics, University of South Florida, Tampa, FL 33620, United States of America; 2 Department of Neurobiology and Behavior, University of California Irvine, Irvine, CA 92697, United States of America; 3 Theoretical Biology and Biophysics, Los Alamos National Laboratory, Los Alamos, NM 87545, United States of America; University of Hull, UNITED KINGDOM

## Abstract

Amyloid beta (Aβ) oligomers associated with Alzheimer’s disease (AD) form Ca^2+^-permeable plasma membrane pores, leading to a disruption of the otherwise well-controlled intracellular calcium (Ca^2+^) homeostasis. The resultant up-regulation of intracellular Ca^2+^ concentration has detrimental implications for memory formation and cell survival. The gating kinetics and Ca^2+^ permeability of Aβ pores are not well understood. We have used computational modeling in conjunction with the ability of optical patch-clamping for massively parallel imaging of Ca^2+^ flux through thousands of pores in the cell membrane of *Xenopus* oocytes to elucidate the kinetic properties of Aβ pores. The fluorescence time-series data from individual pores were idealized and used to develop data-driven Markov chain models for the kinetics of the Aβ pore at different stages of its evolution. Our study provides the first demonstration of developing Markov chain models for ion channel gating that are driven by optical-patch clamp data with the advantage of experiments being performed under close to physiological conditions. Towards the end, we demonstrate the up-regulation of gating of various Ca^2+^ release channels due to Aβ pores and show that the extent and spatial range of such up-regulation increases as Aβ pores with low open probability and Ca^2+^ permeability transition into those with high open probability and Ca^2+^ permeability.

## Introduction

Increased production of the self-aggregating form of amyloid beta (Aβ) peptide caused by abnormal processing of amyloid precursor protein (APP) is a hallmark of Alzheimer’s disease (AD) pathogenesis [1–3]. The imbalance between Aβ production and clearance has been proposed to lead to formation of Aβ plaques [1,4]. More recent discoveries implicate the soluble form of Aβ oligomers, rather than the plaques, as the toxic specie, mediating its effects by disrupting the integrity of cells plasma membrane leading uncontrolled fluxes of Ca^2+^ into the cells [5–10]. Various mechanisms have been proposed to underlie the increased membrane permeability to Ca^2+^, including interaction with several endogenous Ca^2+^ permeable channels [11–15], but studies in lipid bilayer systems [16–18] and in *Xenopus* oocytes [19,20] which lack native Ca^2+^ permeable channels point to the formation of intrinsic Aβ Ca^2+^-permeable pores in the cell membrane as a major mechanism.

The resulting disruption of Ca^2+^ signaling, in turn, has the potential to alter cell function in several ways [12,21,22]. For example, mitochondrial Ca^2+^ overload could cause the loss of mitochondrial membrane potential leading to impaired cell bioenergetics. Importantly, exaggerated intracellular Ca^2+^ concentration has been shown to affect memory formation either by suppressing long-term potentiation or up-regulating long-term depression [21–26].

Neuronal Ca^2+^ signaling dysfunctions are thus proposed to play a key role in AD (see for example, [12,21,22,27,28]). A complete understanding of Ca^2+^ signaling remodeling and toxicity is therefore crucial for both the etiology of AD and designing efficient therapeutic approaches. As a key element in the Ca^2+^ signaling deregulation in AD, elucidating the kinetics of Aβ pore is of a paramount importance for further progress in this area [29,30]. In this paper, we use computational modeling in conjunction with TIRF-based massively-parallel fluorescence imaging of Ca^2+^ flux through individual Aβ pores, to gain insight into the functioning of Aβ pores.

Our ability to simultaneously and independently image Ca^2+^ flux through thousands of channels provides a uniquely advantageous model to investigate Aβ pore functioning [19,20]. In particular, previous findings from our lab revealed enormous variability in open probability (P_O_) and permeability to Ca^2+^ among different Aβ pores, implicating important differences among Aβ pores in terms of their relative contributions toward cellular Ca^2+^ toxicity. Moreover, once inserted in the membrane, time-dependent changes in pore gating properties suggest further rearrangement and aggregation of Aβ oligomers after they incorporate into the cell membrane [19].

Here we use a maximum likelihood-based method developed for separating signal from noise-corrupted drifting background [31] to convert experimental time-series fluorescence records from multiple, individual Aβ pores into idealized traces representing the state in which a pore is conducting at a given time. We extract the statistical properties of the Aβ pores from these idealized traces to develop Markov chain models, so as to better understand the gating properies, P_O_, and permeability of Aβ pores, and how Ca^2+^ influx through the pores may interact with and disrupt cellular Ca^2+^ signaling pathways in AD.

## Methods

### Experimental methods

Detailed experimental methods for imaging Aβ pore activity are described in [19] and are outlined as follows.

Preparation and characterization of Aβ42 oligomers: Soluble oligomers were prepared by dissolving 0.5 mg of human recombinant Aβ42 peptide (hexafluoroisopropanol pretreated; rPeptide) in 20 μl of freshly prepared DMSO and were quickly diluted with 480 μl of double distilled water in a siliconized Eppendorf tube. After a 10 min sonication, samples were incubated at room temperature for 10 min and then centrifuged for 15 min at 14,000 g. The supernatant fraction was transferred to a new siliconized tube and stirred at 500 rpm using a Teflon-coated microstir bar for 8–48 h at room temperature. In our hands, this aggregation method has consistently generated active Aβ42 species consisting of Aβ42 aggregates ranging from 5-to 40-mers. Similar method, water incubation of Aβ42 for equivalent period of time, has been reported to generate toxic Aβ42 soluble oligomers also by other groups [32,33].

Oocyte preparation and electrophysiology: Experiments were performed on defolliculated stage VI oocytes obtained from Ecocyte Bioscience US LLC (Austin, Texas). Oocytes were injected **~**1 h before imaging with fluo-4 dextran (molecular mass of **~**10 kD and Ca^2+^ affinity of **~**3 μM; F.C, ~40 μM). For TIRF microscopy experiments, oocytes were first stripped the vitelline envelope and placed animal hemisphere down in a chamber whose bottom is formed by a fresh ethanol-washed microscope cover glass (type-545-M; Thermo Fisher Scientific) and were bathed in Ringer’s solution (110 mM NaCl, 1.8 mM CaCl_2_, 2 mM KCl, and 5 mM Hepes, pH 7.2) at room temperature (**~**23°C) continually exchanged at a rate of **~**0.5 ml/min by a gravity-fed superfusion system. The membrane potential was clamped at a holding potential of 0 mV using a two-electrode voltage clamp (Gene Clamp 500; Molecular Devices) and was stepped to more negative potentials (-100 mV) when imaging Ca^2+^ flux through amyloid pores to increase the driving force for Ca^2+^ entry in to the cytosol. Solutions containing Aβ42 oligomers were delivered from a glass pipette positioned near the edge of the membrane footprint of the oocyte membrane on the cover glass.

**Fig 1:**
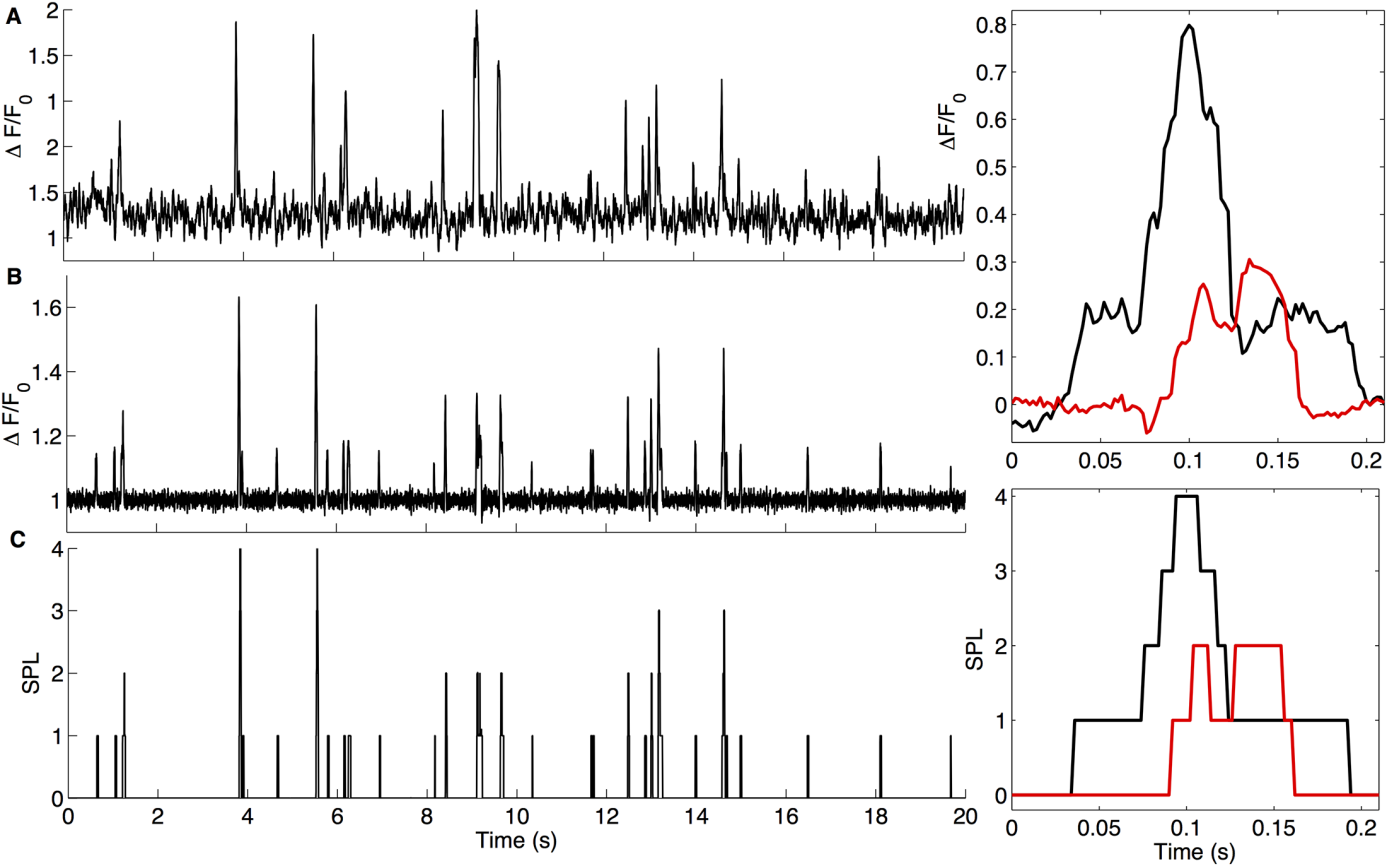
Idealizing the fluorescence time-series data from a single Aβ pore. Fluorescence profile from a region of interest (1μm^2^) centered on an Aβ42 pore, representing the Ca^2+^ flux through the pore without (A) and with background subtraction (B). (C) Idealized trace, where the vertical axis represents the SPL to which the pore opens at a given time and zero represents the closed state. The two insets show two events on extended time-scale (Ca^2+^ flux through the pore with background subtraction (top) and idealized traces (bottom)).

TIRF microscopy, image acquisition, and processing: Imaging was accomplished by using a custom-built TIRF microscope system based around a microscope (IX70; Olympus) equipped with a 60× TIRF microscopy objective (1.45 NA; Olympus; [19,34,35]). Fluorescence excited by a 488 nm laser was imaged using an electron-multiplied charge-coupled device camera (Cascade 128+; Roper Scientific) at full resolution (128 × 128 pixel; 1 pixel = 0.33 μm at the specimen) at a rate of 500 s^-1^. Image data were acquired using the Meta-Morph software package (Universal Imaging) and were black-level corrected by subtracting the camera offset. To compensate for differential time-dependent changes in basal fluorescence across different locations in the image field, a strongly smoothed (10 × 10–pixel Gaussian blur) copy of each frame was calculated to create a running baseline (F_0_) image. The raw image stack was then divided frame-by-frame by the smoothed copy to create a baseline-corrected pseudo-ratio stack in which each pixel represents localized differences in fluorescence relative to the spatially averaged baseline fluorescence (**Δ**F/F_0_). A previously developed Java-based program, CellSpecks was used to identify sites of pore activity, and traces of fluorescence versus time such as those in Fig 1 (top panel) were obtained as the maximum pixel intensity within fixed 3 × 3 pixel (**~**1 × 1 μm) regions of interest centered on putative pore locations [19]. The maximum observed fluorescence signals were small (maximum ΔF/F_o_ < 2.0) in comparison with the full dynamic range of fluo-4 (**Δ**F/F_o_ > 30 in saturating Ca^2+^) and are thus expected to be linearly proportional to Ca^2+^ flux.

### Computational methods

Model selection: The open-source software QUB [36] was employed to fit Markov chain models to the idealized traces (see below for idealization) representing the Ca^2+^ permeability level in which the pore is gating as a function of time. As shown in Fig 1, the pore clearly exhibits gating behavior on the millisecond timescale, which is much faster than the minutes timescale for the pore formation. Thus, the on and off Ca^2+^ flux is due to the opening and closing of the pore rather than its appearance and disappearance, making Markov chains a suitable choice for modeling the gating kinetics of the pore. We developed two separate Markov chain models for each type (based on the maximum permeability that pore can achieve, see below) of pore: (1) the simplest possible model and (2) the best model according to the Bayesian Information Criterion (BIC) [37]. BIC is given by -2 × *ln*(maximized value of *Likelihood*) + *k × ln*(*N*), where *ln*, *k*, and *N* respectively represent natural log, the total number of parameters in the model, and total number of data points used in fitting. The BIC score penalizes for the number of parameters. Thus increasing the *Likelihood* score by adding more parameters (states and connections) doesn’t necessarily mean low (better) BIC score as the penalty for the number of extra parameters in the model might be higher than the gain in *Likelihood*.

The log of the Likelihood function for the current traces is given as [38]

$$\begin{array}{ll} ln(f(t_{C1},t_{O1},t_{C2},t_{O2},.....t_{Cn}))= & ln(\pi_{C}exp(Q_{CC}t_{C1})Q_{CO}exp(Q_{OO}t_{O1})\\ & Q_{OC}exp(Q_{CC}t_{C2})Q_{CO}exp(Q_{OO}t_{O2})...\\ & exp(Q_{OO}t_{On})u_{O} \end{array}$$

where *π*_*C*_ is the initial probability of closed states being occupied at equilibrium, *t*_*Oi*_ and *t*_*Ci*_ are the *ith* opening and closing in the time-series respectively, and *Q*_*CC*_, *Q*_*OO*_, *Q*_*OC*_, and *Q*_*CO*_ are the sub-matrices of the N×N generator matrix *Q* of the Markov chain with N states. That is,

$$Q=\left(\begin{array}{cc} Q_{OO} & Q_{OC}\\ Q_{CO} & Q_{CC} \end{array}\right)$$

The element of *Q* at location *ij*, *Q*_*ij*_; *i* × *j* is the transition rate from state *i* to state *j*. The diagonal entries are given by *Q*_*ii*_ = −Σ_*j*≠*i*_ *Q*_*ij*_, which is an expression of conservation of probability [39]. Thus *Q*_*CC*_, *Q*_*OO*_, *Q*_*OC*_, and *Q*_*CO*_ are matrices of the transition rates from all closed to all closed, all open to all open, all open to all closed, and all closed to all open states, respectively. If a model has *N*_*C*_ closed and *N*_*O*_ open states, the *Q*_*CC*_, *Q*_*OO*_, *Q*_*CO*_, and *Q*_*OC*_ are *N*_*C*_ x *N*_*C*_, *N*_*O*_ x *N*_*O*_, *N*_*O*_ x *N*_*C*_, and *N*_*C*_ x *N*_*O*_ matrices respectively. For data obtained at equilibrium, π_*C*_ = *W*_*O*_ *Q*_*OC*_ / *J* with *J = W*_*O*_ *Q*_*OC*_ *uC*, where *u*_*C*_ is an *N*_*C*_—component vector of all 1's. *W*_*C*_ and *W*_*O*_ are diagonal matrices of the equilibrium occupancies of all closed and all open states respectively.

The total log-likelihood of all data used in the fit is calculated as

$$ln(likelihood(data))=\sum_{i=1}^{N_{exp}}ln(likelihood(data_{i})).$$

Where *N*_*exp*_ is the number of experiments and *data*_*i*_ is the data set (time series) from experiment *i*.

The simplest model that we develop has one closed state and one open state each for a sub-permeability level (SPL) (SPL is analogous to sub-conductance level except that here the sub-levels are in term of Ca^2+^ permeability (flux) rather than conductance). Thus, the number of states in the simplest model is equal to 1 + maximum SPL (SPL_M_) that the pore can open to. For the best model, we started with the simplest model and kept adding states till the BIC score stopped decreasing and chose the model with the lowest (best) BIC score. For example, the simplest model for the type 1 pore (pore with one closed and one open level (one SPL)) has only two states, one closed and one open. To search for the best model, we added a second closed state to the simplest model and allowed QUB to minimize the BIC score by optimizing the transition rates and testing for all the connections allowed by the data (see below). Similarly, we added a second open state to the simplest model and minimized the BIC score. Of the two additional states we chose the one that gave the lowest BIC score. We repeated this procedure till the best BIC score was reached. We found that in most cases the model with best BIC score also gave the best (smallest) Akaike Information Criterion (AIC) score [37].

Detail balance: In S1 Text we show that our data are consistent with the microscopic reversibility hypothesis (see also S1 Fig). In the light of this observation, we will ignore all those models where some loops violate detailed balance.

Stochastic modeling of Aβ pores: Traces representing the gating state of the pore and the changes in Ca^2+^ concentration ([Ca^2+^]) due to Aβ42 pore opening as a function of time were simulated using the procedure in [40,41]. Detail of stochastic scheme of the pore and diffusion of Ca^2+^ in presence of dye buffer is given in the S1 Text.

Open probability of IP_3_R, Ca^2+^-activated Cl^-^ channel, and BK channels: To estimate the P_O_ of inositol 1,4,5-trisphosphate (IP_3_) receptor (IP_3_R) channel, we adopted our previously published 7-state model that satisfactorily reproduces the P_O_ of type 1 IP_3_R in Xenopus *laevis* oocytes (see S2 Fig of [41]). A similar model explained all observations about IP_3_R in Spodoptera frugiperda (Sf9) cells [42]. The P_O_ of IP_3_R is given by the functional form [41]

$$Po=\frac{K_{O24}{\left[Ca^{2+}\right]}^{2}{\left[IP_{3}\right]}^{4}}{Z}$$ (1)

Where [*IP*_*3*_] is the IP_3_ concentration and

$$Z=K_{R00}+K_{A20}{\left[Ca^{2+}\right]}^{2}+K_{I50}{\left[Ca^{2+}\right]}^{5}+K_{R04}{\left[IP_{3}\right]}^{4}+K_{A24}{\left[Ca^{2+}\right]}^{2}{\left[IP_{3}\right]}^{4}+K_{O24}{\left[Ca^{2+}\right]}^{2}{\left[IP_{3}\right]}^{4}+K_{I54}{\left[Ca^{2+}\right]}^{5}{\left[IP_{3}\right]}^{4}$$

*K*_*X*_[*Ca* ^*2+*^]^*m*^[*IP*_*3*_]^*n*^ in the above equations is the occupancy of various states at given [*Ca* ^*2+*^] and [*IP*_*3*_] values. The model has one open state, O_24_, with two Ca^2+^ and four IP_3_ bound, and six closed states, R_00_, A_20_, I_50_, R_04_, A_24_, and I_54_, where the two numbers in the subscript represent the number of Ca^2+^ ions and IP_3_ molecules bound in that state. The occupancy parameters are: K_R00_ = 1, K_A20_ = 1.035×10^5^ μM^-2^, K_I50_ = 1.0×10^4^ μM^-4^, K_R04_ = 3.11×10^10^ μM^-4^, K_A24_ = 1.135×10^11^ μM^-6^, K_O24_ = 4.56×10^11^ μM^-6^, K_I54_ = 2.296×10^6^ μM^-9^ (see [41] for further details about the IP_3_R channel model).

The normalized current through Ca^2+^-activated Cl^-^ channel as a function of Ca^2+^ concentration is modeled by Hill equation

$$I_{Norm}=I_{max}\frac{1}{{\left(K_{D}/[Ca^{2+}]\right)}^{n}+1}$$ (2)

Where the parameters *I*_*max*_ = 1.0 (normalized to a peak current of 250pA), *K*_*D*_ = 2.2μM, and *n* = 2.8 were obtained by fitting eq (2) to the current through Ca^2+^-activated Cl^-^ channel in rat olfactory receptor neurons at various [Ca^2+^] values and membrane potential, V = -40mV (Fig 4B in [43]).

The P_O_ of big K^+^ (BK) channel is also given by Hill equation

$$P_{O}=P_{max}\frac{1}{{\left(K_{D}/[Ca^{2+}]\right)}^{n}+1}$$ (3)

Where two sets of parameters *(P*_*max*_ = 0.27, *K*_*D*_ = 35μM, *n* = 1.98) and *(P*_*max*_ = 0.95, *K*_*D*_ = 3.3μM, *n* = 2.25) were obtained by fitting eq (3) to the P_O_ data from BK channel in cerebellar Purkinje neurons at V = - 60mV and 40mV respectively (Fig 6A in [44]).

## Results

### Idealizing single-pore Ca^2+^ fluorescence traces

Modeling the gating kinetics of a pore requires idealized Ca^2+^ dependent fluorescent traces representing a relative measure of Ca^2+^ permeability or Ca^2+^ flux (Ca^2+^ current) through the pore. First, all traces are filtered using a moving box average method with a window of 5 points. Then to idealize the resultant time-series traces from individual pores, we extend our Maximum *Likelihood*-based method developed for separating signal from baseline in noisy quantal electrophysiological data [31] to the TIRF fluorescence data. The method involves the removal of drifting baseline and noise from the traces using expectation-maximization (EM) algorithm. The full detail of the method and the relevant software is given [31]. Briefly, we treat the observed fluorescence data as comprising three components: (1) a quantal flux (current) passing through the pore, (2) a slowly varying baseline signal that we model as a random walk, and (3) a white noise that represents noise within the measurement apparatus (note that the method works equally well for non-white noise). We denote the fluorescence data (ΔF/F_0_) record from discrete sampling by *d* = (*d*_*1*_, *d*_*2*_, *…*.., *d*_*T*_) and a generic time point in the record by *d*_*t*_, where *t*∈(*1*,*2*, ….., *T*). Assume that an open pore passes Ca^2+^ flux in units of *f* and a closed pore passes no flux. If the pore is open up to SPL, *n*_*t*_, at time t, the observed signal (the data, *d*_*t*_) is given by *d*_*t*_ = *b*_*t*_ *+ f × n*_*t*_ *+ σ*_*ξ*_ *× ξ*_*t*_, where *b*_*t*_ is the baseline flux although not necessarily through the pore, and *ξ*_*t*_ is discrete-time Gaussian white noise that has moments *<ξ*_*t*_ *> = 0*, *<ξ*_*t*_ *ξ*_*t*_^*/*^ *> = δ*_*tt*_^*/*^. Here *δ*_*tt*_^*/*^ is the Kronecker delta and *σ*_*ξ*_ is the noise strength. We further assume that the baseline is undergoing a discrete time random walk, *b*_*t*_ = *b*_*t-1*_ *+σ*_*b*_ *× ξ*_*t*_ where *ξ*_*t*_ is discrete-time white noise like *ξ*_*t*_. Under these assumptions, it follows immediately that *d*_*t*_ *—b*_*t*_ *—f × n*_*t*_ and *b*_*t+1*_—*b*_*t*_ are zero mean Gaussian distributed random variables with variances *σ*_*ξ*_^2^ and *σ*_*b*_^2^ respectively. Analogous to the definition of *d*, we also define *b* and *n*: *b = (b*_*1*_, *b*_*2*_, *…*.., *b*_*T*_ *)*, *n = (n*_*1*_, *n*_*2*_, *…*..*n*_*T*_ *)*. Our primary goal is to obtain estimates of the *n*_*t*_, but in doing so we will also obtain estimates of *b*, *σ*_*ξ*_^2^, *σ*_*b*_^2^, and *f* as well. It turns out that it is simpler to parameterize the system in terms of *σ*_*ξ*_^2^ and *R* ^*2*^, where *R* ^*2*^ = *σ*_*b*_^2^/*σ*_*ξ*_^2^ is the ratio of the baseline walk variance to the white-noise variance. We treat both *n* and *b* as missing data, although *b* is integrated out directly without the need of EM. Our treatment of the signal implies that the joint distribution function for *d*, *b*, and *n* is given by

$$p(d,b,n;\theta )=\tilde{N}e^{-H(d,b,n;\theta )}$$ (4)

Where *H* is given by

$$H=(d,b,n;\theta )=\frac{1}{2\sigma_{b}^{2}}\sum_{t=1}^{T-1}{\left(b_{t+1}-b_{t}\right)}^{2}+\frac{1}{2\sigma_{\xi }^{2}}\sum_{t=1}^{T-1}{\left(d_{t}-b_{t}-f\times n_{t}\right)}^{2}$$ (5)

and *θ* represents the parameters *σ*_*ξ*_^2^, *σ*_*b*_^2^, and *f*. The value *Ñ* is the normalization factor. The rest is to estimate *n*_*t*_ (along with other parameters) by maximizing the *ln*(*Likelihood*) of the distribution function in eq (4) using EM algorithm. The idealized traces represent the SPL in which the pore is gating as a function of time.

Fig 1A plots the average fluorescence within a 1 x 1 μm region centered on a pore from an image sequence recorded at a sampling rate of 500 frames per second. Fig 1B and 1C show, respectively, the background and noise corrected, and idealized fluorescence time-series trace derived from this record.

A detailed analysis and the reasons for us to believe that the Ca^2+^ dependent fluorescence fluctuations such as shown in Fig 1A are due to the gating of Aβ pores is given in a previous paper [19]. Among the reasons are: (1) the localized “channel like” fluorescence flickering was not present in any of the control oocytes and they appear only after the oocytes were incubated with Aβ42 oligomers, not monomers; (2) The single channel activity was blocked by zinc which is known for blocking Aβ pores; and (3) The single channel behavior in our experiments is very similar to the behavior reported for Aβ pore from lipid bilayer studies [16–18]. Further details about the physiological characterization of the Aβ pores, reproducibility, and control data are given in a previous paper [19].

The maximal fluorescence signal in the example in Fig 1 corresponds to opening to a maximum of four SPLs. After idealizing 20s long time-series traces from 643 pores, we observed pores opening up to a maximum of five SPLs. For a given pore, we consider the peak Ca^2+^ permeability (in units of permeability level) observed in a trace to be the maximum permeability that the pore can achieve. Traces were separated into five groups, for pores having a maximum permeability of up to 1 (SPL_M_ 1), 2 (SPL_M_ 2), 3 (SPL_M_ 3), 4 (SPL_M_ 4), and 5 (SPL_M_ 5) SPLs. For example the trace shown in Fig 1C was included in the group with SPL_M_ 4. For the rest of the paper, pores with SPL_M_ 1, SPL_M_ 2, SPL_M_ 3, SPL_M_ 4, and SPL_M_ 5 are referred to as type 1, 2, 3, 4, and 5 respectively.

We analyzed single-pore imaging data from 484 active pores in three oocytes at time-point of 20, 25, and 30 minutes each after adding the solution containing Aβ oligomers. Notice that the three oocytes used for the modeling here were selected from our previous study where we characterized the formation and activity of Aβ pores performing many experiments on over 30 oocytes [19]. Aβ pores in all experiments revealed consistent gating kinetics and the data used for modeling was representative of all those experiments. Moreover, our optical patch-clamp technique is capable of recording thousands of channels simultaneously and independently providing massively parallel tools to gather information on ion channels behavior. Specifically for the data reported in this paper each 20 seconds record contain information on about 1000 channels simultaneously and independently. This is the equivalent of 33 minutes of single channel recording obtained using conventional electrical patch clamp technique.

**Fig 2:**
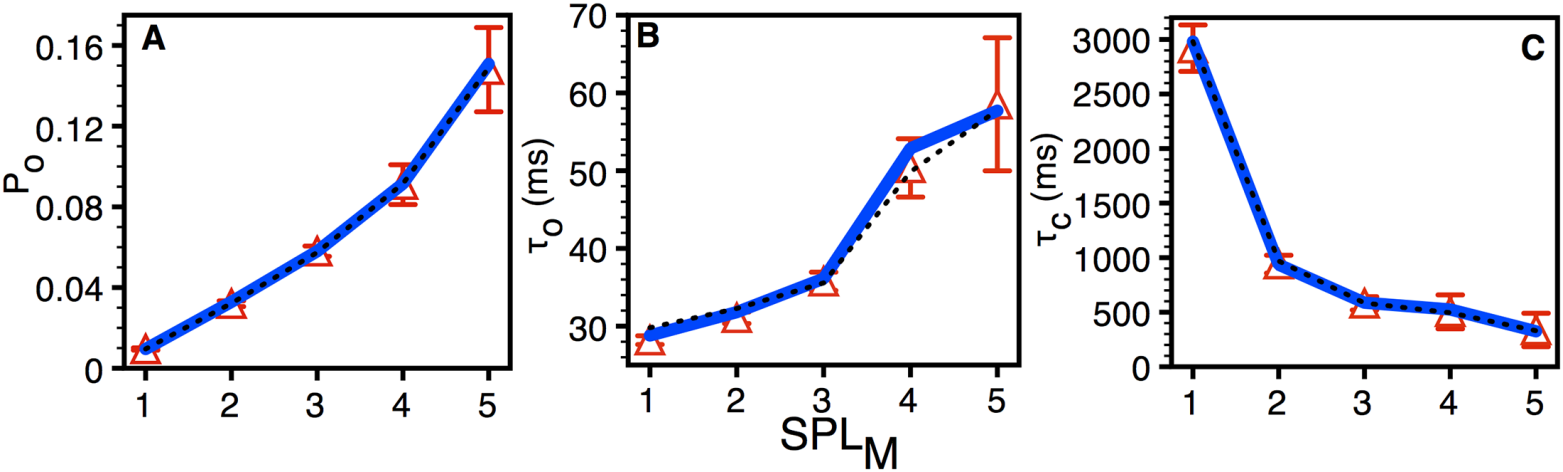
Average statistics of Aβ42 pores. Mean open probability (A), open time (B), and closed time (C) as a function of SPL_M_ (maximum permeability in units of SPLs) for all pores. The symbols represent the experimental data. The thick solid and thin dotted lines are the fits from the simplest and best models respectively presented below. The bars represent standard error of the mean.

The number of pores exhibiting relatively high permeability and hence P_O_ (as pores with higher permeability have higher P_O_, see Fig 2 below) is increased with time after incubation (Table 1) [19]. The number of type 1 and 2 pores decreased significantly with time (Table 1), whereas the numbers of pores with higher conductance (types 3–5) on the other hand increased with time after incubation. This behavior led Demuro et al. [19] to hypothesize that individual pores may show a slow, progressive increase in maximal permeability level (e.g. a pore might transition from type 4 to type 5 over several minutes), possibly due to additional Aβ monomers or small oligomers. However, we were unable to reliably detect such transitions during the limited duration (20s) of each imaging stack, and separately consider and model pores of each type (but see Discussion section).

**Table 1:** The number of pores of type 1 (N1), type 2 (N2), type 3 (N3), type 4 (N4), and type 5 (N5) observed within a 10 x 10 μm region of three oocytes as a function of time (in minutes) after addition of Aβ oligomers to the oocytes. First column shows the time after addition of Aβ oligomers at which the 20s fluorescence traces were obtained from 484 active pores.

Time (min)	N1	N2	N3	N4	N5
20	218	187	79	0	0
25	173	146	134	24	7
30	116	151	169	35	13

The statistical properties of Aβ pores averaged over 643 pores (20 s traces from 643 pores at single time-point) from the same three oocytes as above are shown in Fig 2. The P_O_ is calculated by dividing the total time for which the pore was open during a record by the duration of the record. The Po correlated strongly with peak permeability (value of SPL_M_) (Fig 2A). This arose primarily because τ_C_ shortened as a strong function of SPL_M_ (Fig 2C), whereas τ_O_ increased only slightly (Fig 2C).

### Modeling the kinetics of the pores

Connectivity of SPLs: Before fitting Markov chain models to the data, we extract the connectivity (probability of transition) of SPLs directly from the idealized data. Note that the connectivity between states having the same permeability cannot be inferred directly from the data without fitting the Markov chain models. Once we know the connectivity of the SPLs, we can ignore the transition between SPLs during the modeling that are not supported by observations. To test for connectivity between various SPLs, we analyzed all transitions in all experimental traces from all pores, recording the initial and final SPLs during the transition. For type 2 pore, the only transitions are among the adjacent permeability levels, i.e. closed level to/from SPL 1 and SPL 1 to/from SPL 2. We found no direct transitions from closed level to SPL 2 within the limits of our sampling rate. For type 3, we found only 1 direct transition from closed level to SPL 2 out of 9165 total transitions. There are no other direct transitions between permeability levels that are more than one level apart, for example, SPL 1 to/from SPL 3 etc. None of type 4 and 5 pores showed any direct transitions between states that are more than one SPL apart.

With the exception of type 3 pore (where we observed one direct transition from closed level to SPL 2), we ignored models with direct transitions between states that are more than one permeability levels apart while searching for the best model for a specific pore type. However, such connections were occasionally added to make sure that they indeed worsened the quality of the fit. All other possible connections among the states with the same conductance and those separated by one permeability level were optimized to reach the best (according to BIC score) connectivity. Despite the fact that the transition probability is very small, we included models with closed-to SPL-2 transition in the model search for type 3 pore.

**Fig 3:**
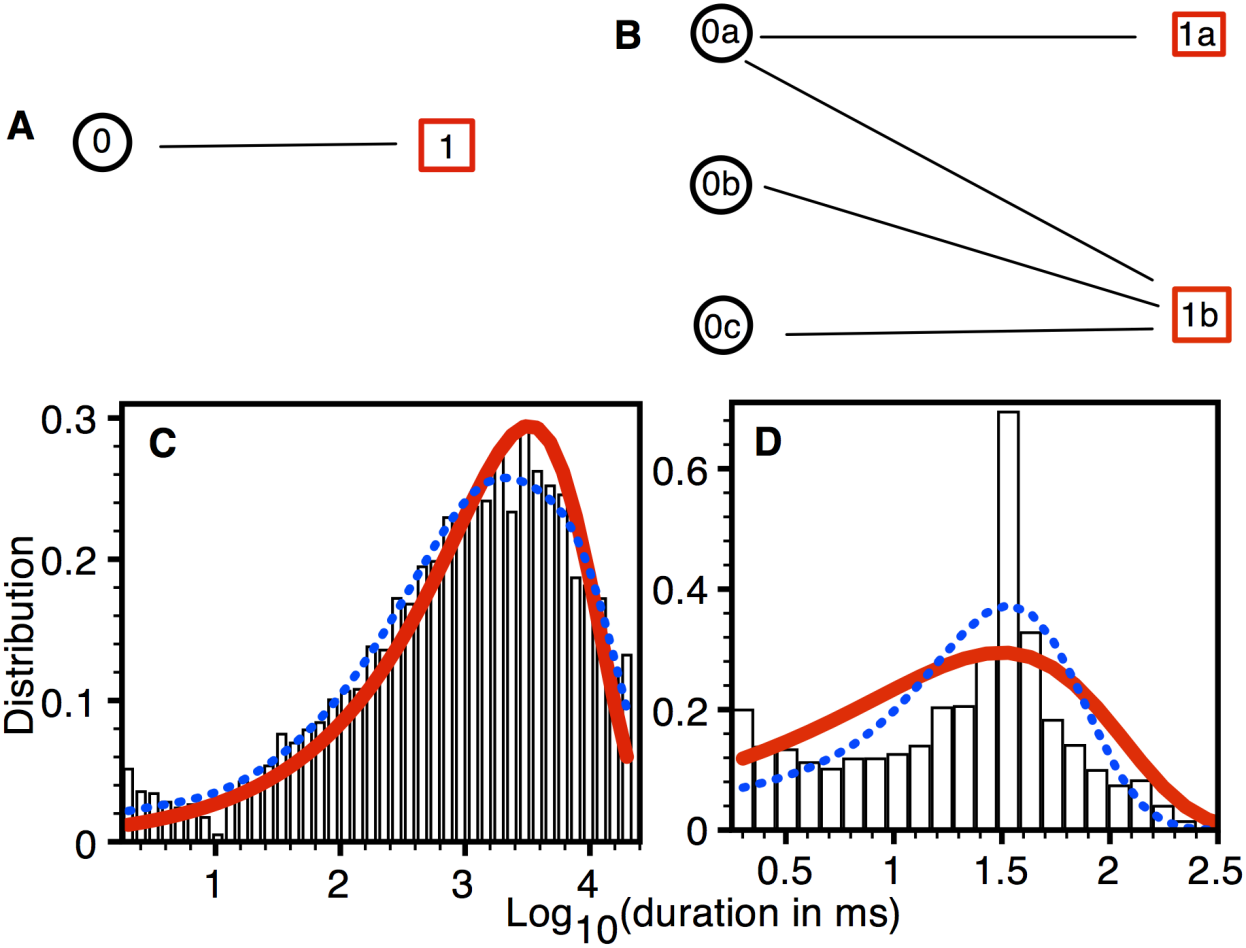
Model fits to the distributions from type 1 pore. (A) The simplest (BIC = -926) and (B) best (BIC = -1071) models. Closed (C) and open (D) dwell-time distributions (probability density functions (PDF)): bars (experimental data), thick solid line (simplest model), and thin dotted line (best model). Experimental data taken from 217 type 1 pores in three oocytes.

Markov Chain Models: Fits to the experimental data from type 1 pores with simplest and best models are shown in Fig 3. As we discussed above, the simplest model only has one state per permeability level, i.e. one state each in closed level and SPL 1. The best model on the other hand is reached by searching for the combination of states and connectivity (topology) and rates that give the smallest BIC score. For remainder of the paper 0, 1 2, 3, 4, and 5 in the name of the state represents closed level, SPL 1, SPL 2, SPL 3, SPL 4, and SPL 5 respectively. While a, b, … is used for multiple states within a given SPL. The transition rates for the models in Fig 3A and 3B are given in S1 Table. As is clear from the figure, the model with best BIC score provides a better fit to the closed (Fig 3C) and open (Fig 3D) dwell-time distributions when compared with those from the simplest model.

**Fig 4:**
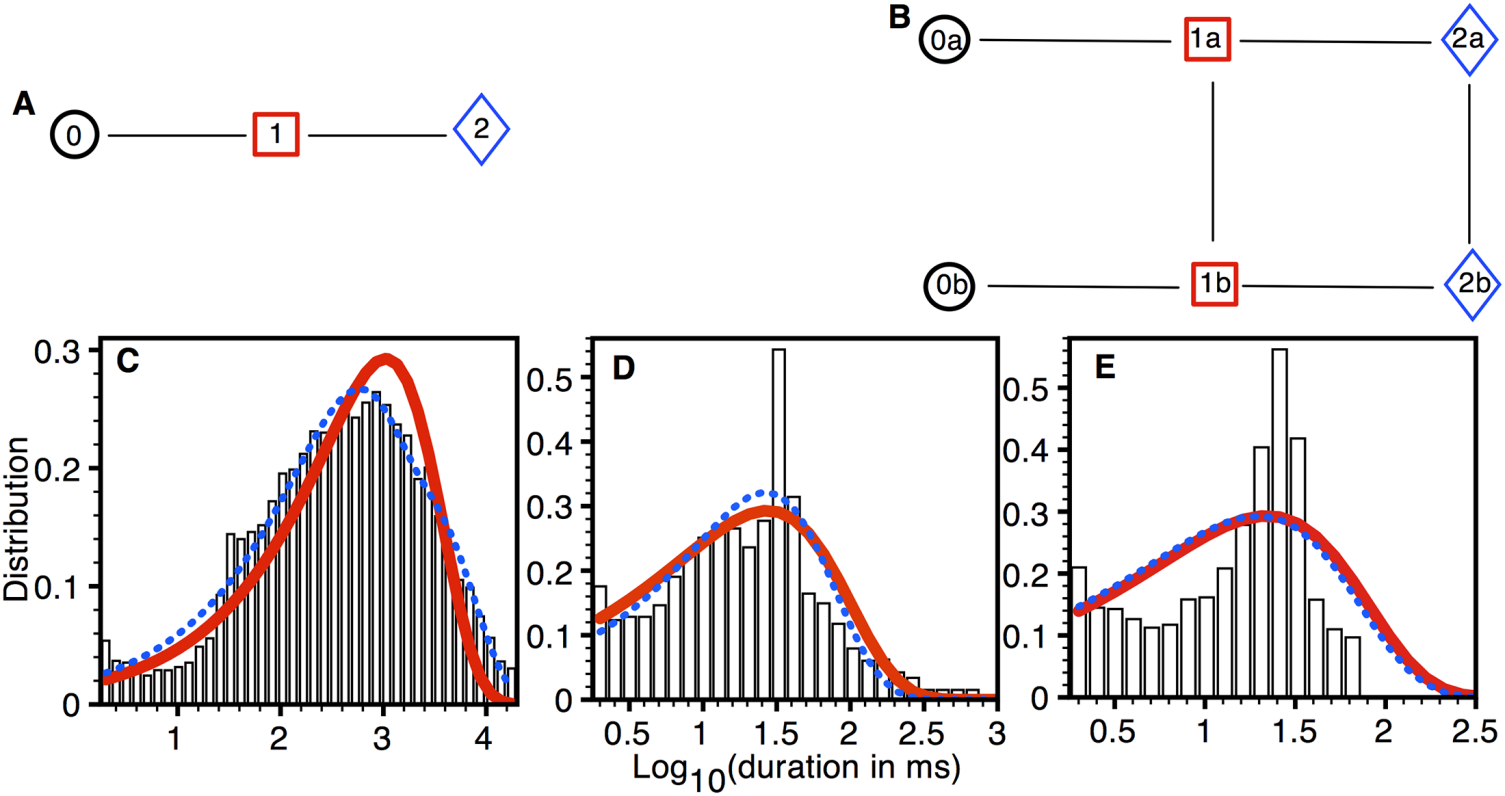
Model fits to type 2 pores. (A) The simplest (BIC = -16495) and (B) best (BIC = -17039) models. Dwell-time distribution (PDF) in closed state (C), SPL 1 (D), and SPL 2 (E): bars (experimental data), thick solid line (simplest model), and thin dotted line (best model). Experimental data taken from 232 type 2 pores in three oocytes.

The model fits to the data from 232 type 2 Aβ42 pores from three oocytes are shown in Fig 4. The simplest model is a linear chain with three states (Fig 4A). As we discussed above, there are no direct transitions between closed level and SPL 2. To ensure that including such transition wouldn’t improve the model, we fit a three state model with cyclic topology (all to all connections) to all traces from type 2 pore. Although the linear chain shown in Fig 4A and cyclic chain had the same *ln*(*Likelihood*) scores, the AIC and BIC scores of the linear chain were smaller (better) as compared to the cyclic chain. Furthermore, the (forward, backward) rates between states 0 and 2 were (8.916×10^−12^, 9.175×10^−10^) as compared to (1.068, 35.48) for the 0↔1 and (5.442, 50.95) for 1↔2 reactions. This confirms that the transitions not allowed by observations indeed worsen the quality of the fit.

A model with the best BIC score was reached by adding two states each for SPL 0, 1, and 2 to the simplest model (Fig 4B). It is possible to improve the *ln*(*Likelihood*) score of the fits to the dwell-time distributions in SPL 1 and 2 (Fig 4C, 4D and 4E) by adding more states to these two SPLs, however that worsens the BIC score as the number of additional parameters over-weighted the improvement in the fits. S2 Table lists the transition rates for the two models shown in Fig 4A and 4B.

**Fig 5:**
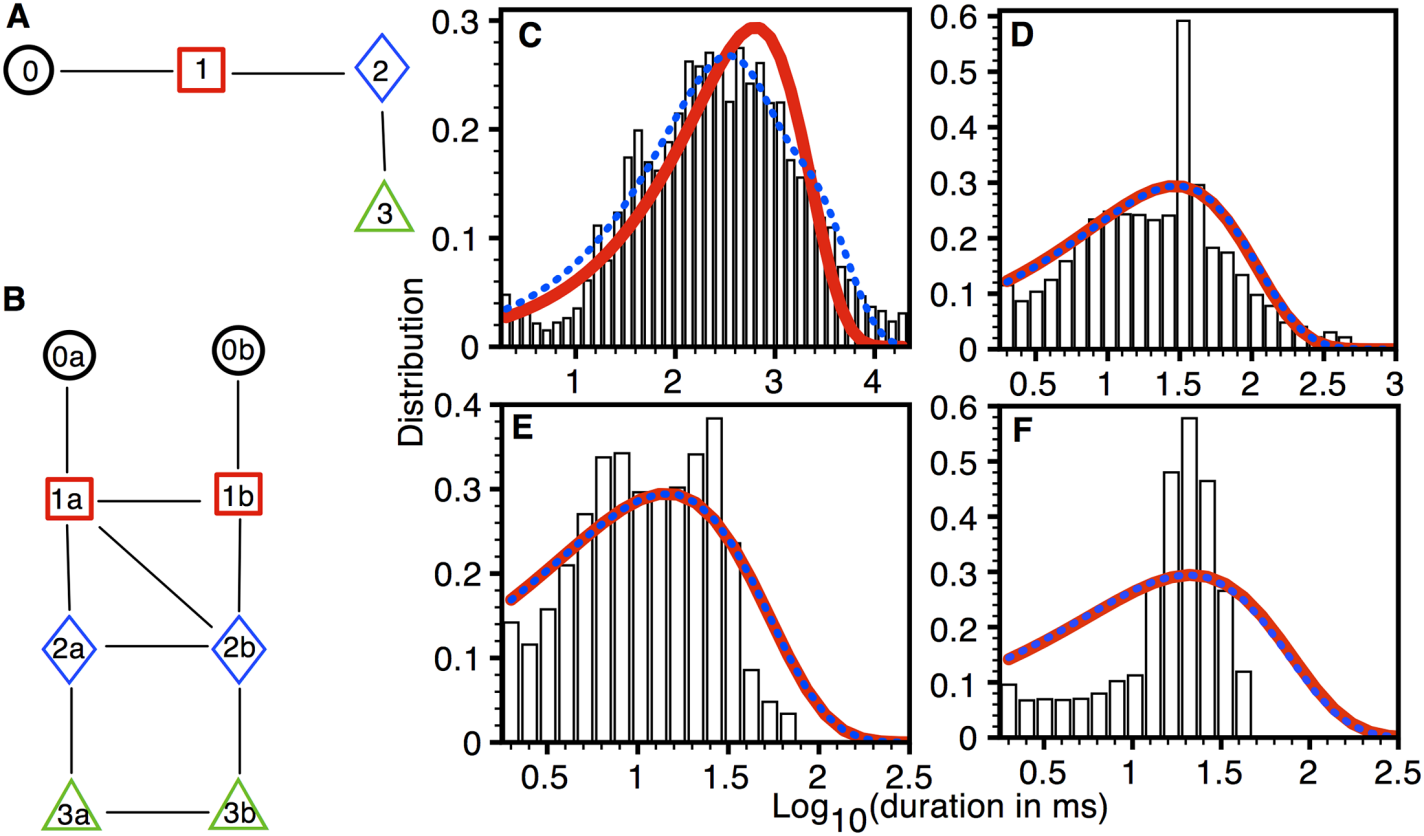
Model fits to type 3 Aβ pores. (A) The simplest (BIC = -21844) and (B) best (BIC = -22887) models. Dwell-time distribution (PDF) in closed level (C), SPL 1 (D), SPL 2 (E), and SPL 3 (F): bars (experimental data), thick solid line (simplest model), and thin dotted line (best model). Experimental data taken from 146 type 3 pores in three oocytes.

As discussed above for the pores that could open up to three SPLs, we found 1 direct transition from closed level to SPL 2 out of 9165 total transitions. Thus, we included models with closed to SPL 2 connections in our search. The simplest and best models that we got by fitting to 146 type 3 pores from 2 oocytes are shown in Fig 5A and 5B respectively. Although, the fits to the dwell-time distributions in different permeability levels (D-F) from both models seem almost similar, the BIC score for the best model was significantly lower than the simplest model. The fit to the lifetime distribution in the closed state from the model with the best BIC score is clearly better than the simplest model (Fig 5C). The transition rates for the two models are given in S3 Table.

**Fig 6:**
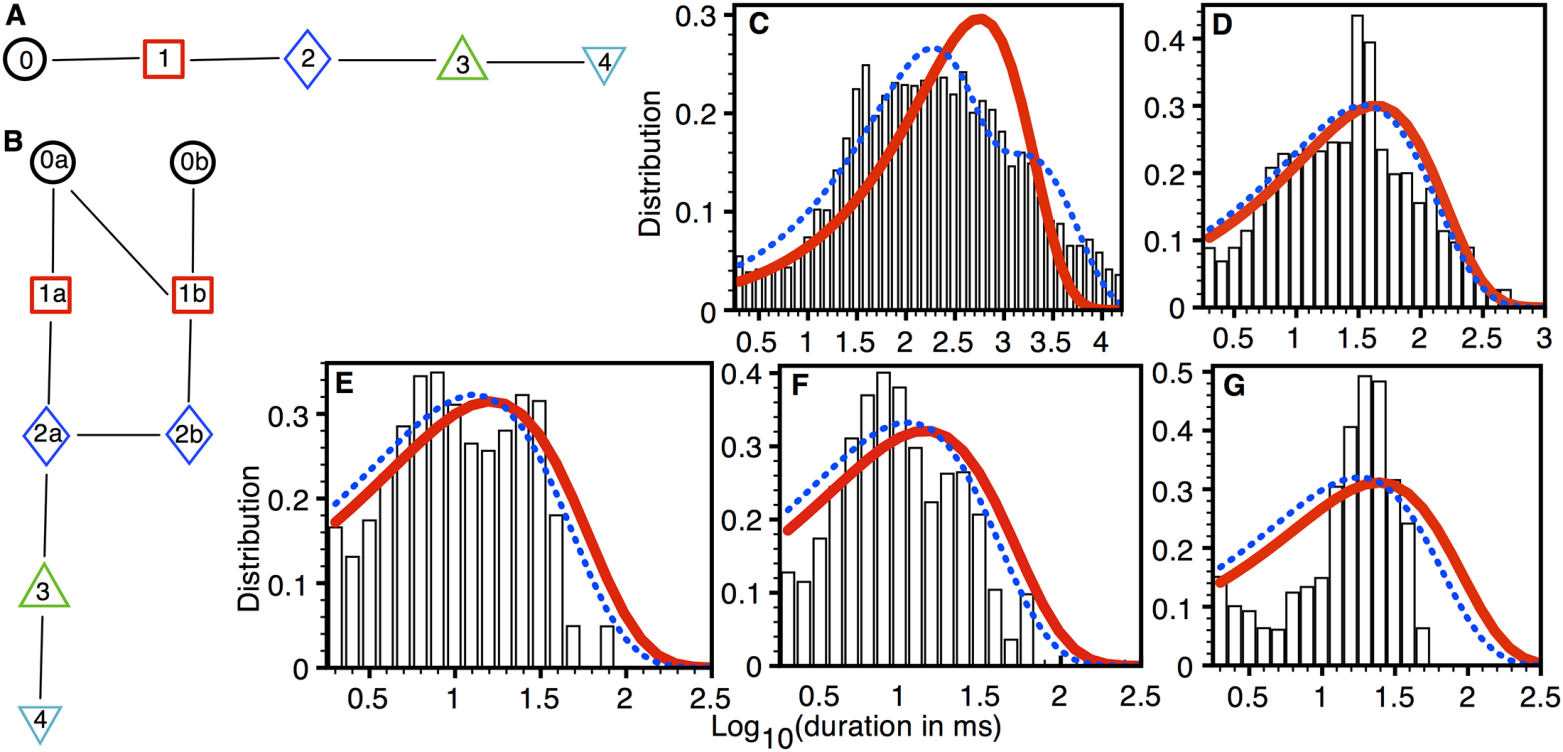
Model fits to type 4 Aβ pores. (A) The simplest (BIC = -8619) and (B) best (BIC = -9449) model. Dwell-time distribution (PDF) in closed level (C), SPL 1 (D), SPL 2 (E), SPL 3 (F), and SPL 4 (G): bars (experimental data), thick solid line (simplest model), and thin dotted line (best model). Experimental data taken from 35 type 4 pores in three oocytes.

Fig 6A and 6B respectively show the simplest and best models for type 4 Aβ1–42 pores. Fluorescence traces were obtained from 35 type 4 pores from 2 oocytes. The observed and model dwell-time distributions in different permeability levels are shown in Fig 6C–6G and the transition rates for the simplest and best models are given in S4 Table.

**Fig 7:**
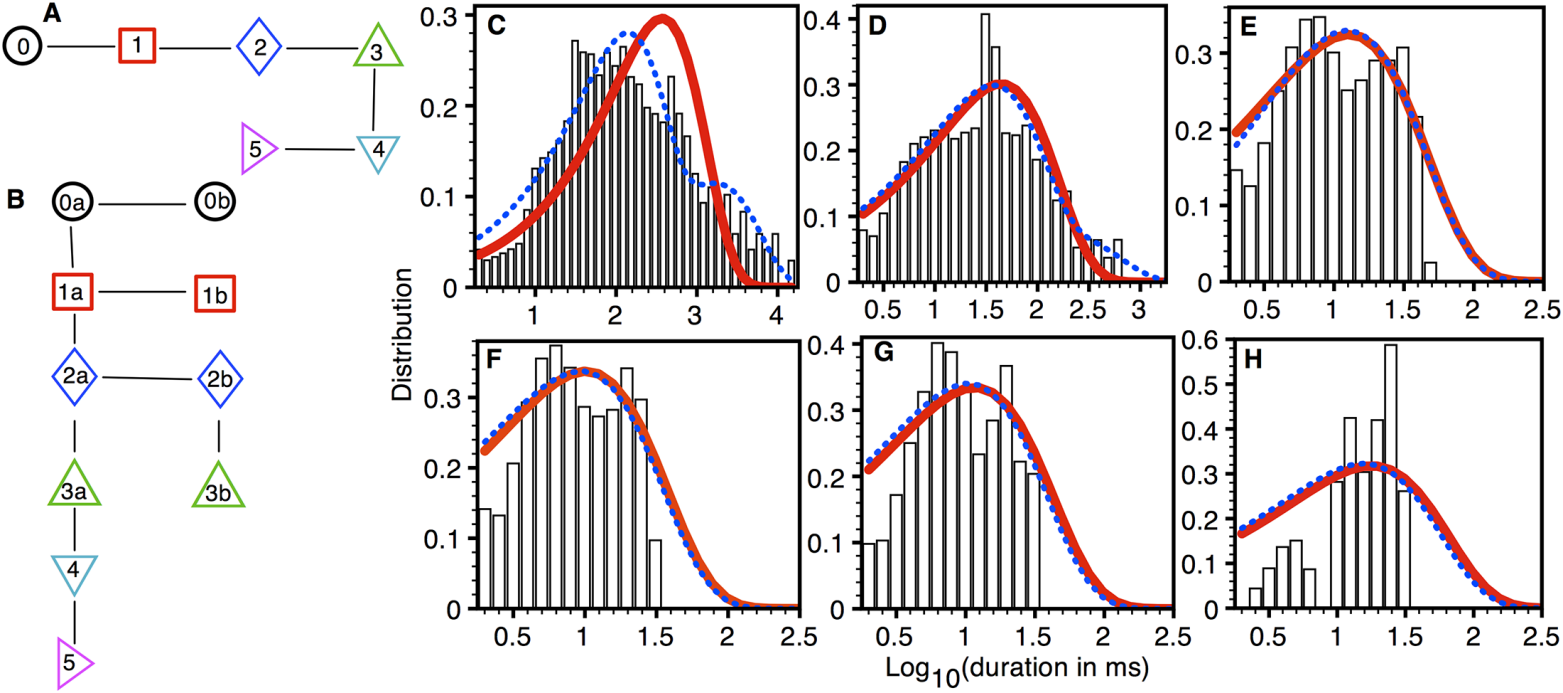
Model fits to type 5 Aβ pores. (A) The simplest (BIC = -4738) and (B) best (BIC = -5229) models. Dwell-time distribution (PDF) in closed level (C), SPL 1 (D), SPL 2 (E), SPL 3 (F), SPL 4 (G), and SPL 5 (H): bars (experimental data), thick solid line (simplest model), and thin dotted line (best model). Experimental data taken from 13 type 5 pores in three oocytes.

Fig 7A and 7B respectively show the simplest and best models fitted to data from 13 type 5 Aβ42 pores from 2 oocytes. Fits to the observed dwell-time distributions in different permeability levels by the simplest and best models are shown in Fig 7C–7H. Four additional states to the simplest model were required to reach the model with best BIC score. As clear from Fig 7C–7H, the decrease in the BIC score by adding more states is mostly due to the improvement in the fit to the dwell-time distributions in the closed level and SPL 1. The rate constants for the simplest and best models are given in S5 Table.

To avoid over-parameterized models, we performed Kienker transformations [45] in order to search for Bauer-Kienker uncoupled (BKU) canonical forms of the models developed above. In the BKU canonical form first described by Bauer et al. [46] and Kienker [45], only the transitions between states having different permeability (conductance) are allowed with no links between states having the same permeability. The detail of Kienker transformations is given in the S1 Text. With the exception of the model in Fig 3B (which is already in BKU form), the BKU canonical forms for all Aβ pore models generally had more connections as compared to the models presented here and also had negative rates between some states. Hence the BKU canonical forms were not considered for further analysis.

**Fig 8:**
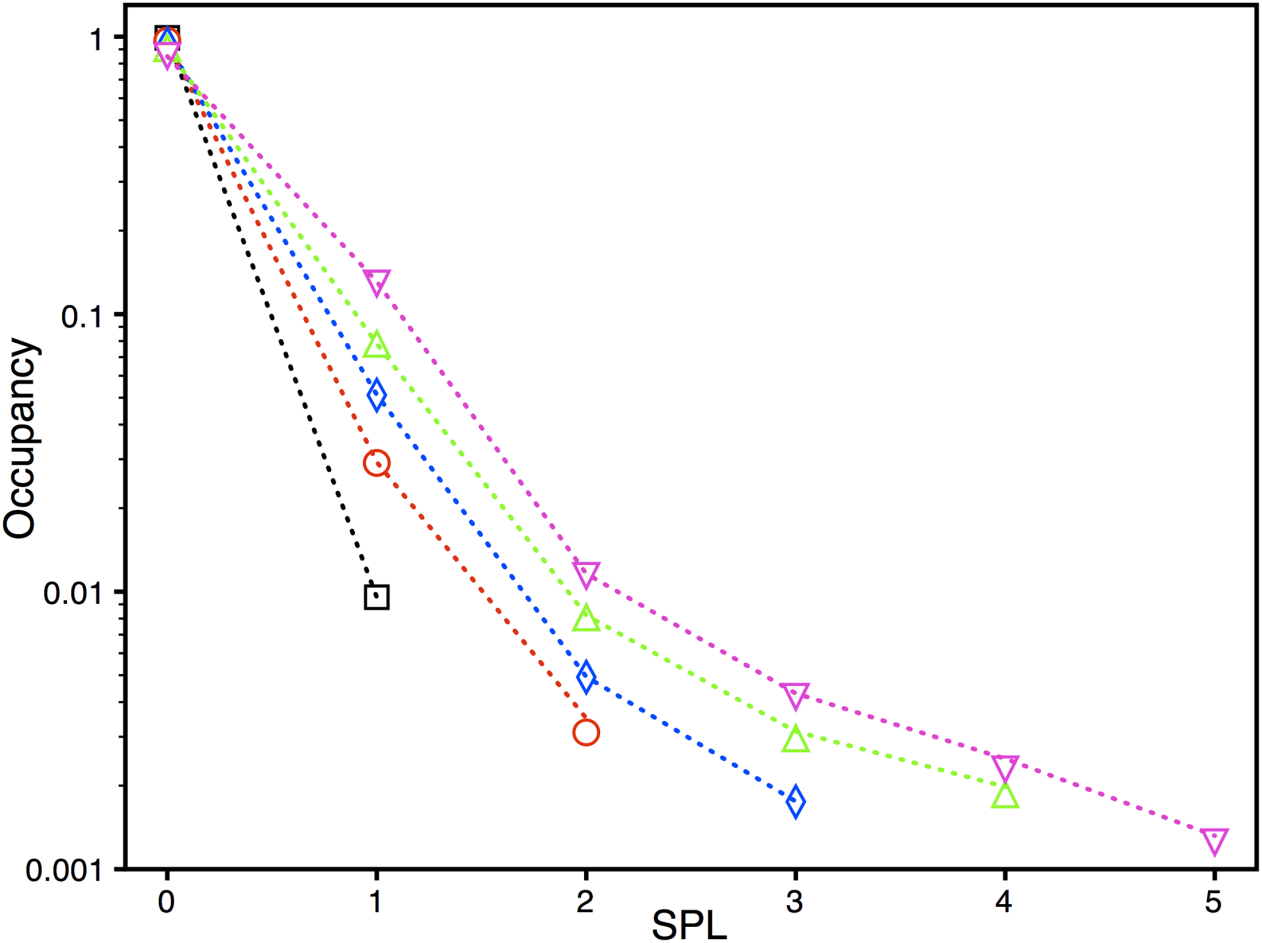
The normalized mean occupancies of all conductance levels in all five groups of Aβ42 pores. The squares, circles, diamonds, up, and down triangles represent the experimental data and dotted lines are the fits from the best models. Black, red, blue, green, and pink represent pores with SPL_M_ 1, 2, 3, 4, and 5 respectively.

Fig 8 shows the normalized mean occupancies (probability of pore in the given permeability level) of all permeability levels in all five types of Aβ42 pores. The models (dotted lines) give excellent fits to the observed occupancies (symbols). It is interesting to note that all types of pores stay for a short time in the highest SPL that the pore can achieve as compared to the lower SPLs. That is, the pores visit the highest SPL that is attainable by a given pore type very briefly. Thus the higher P_O_ observed for pores with higher permeability is mainly due to the longer time that these pores spent in the lower SPLs with very small contribution from highest SPL.

Model time-series traces and Ca^2+^ profiles: Twenty seconds long sample traces representing the permeability level in which the pore is gating as a function of time for the five groups of Aβ42 pores are shown in S2 Fig. In S3 Fig, we compare traces representing Ca^2+^ influx through various Aβ pores as a function of time from experiment and models.

**Fig 9:**
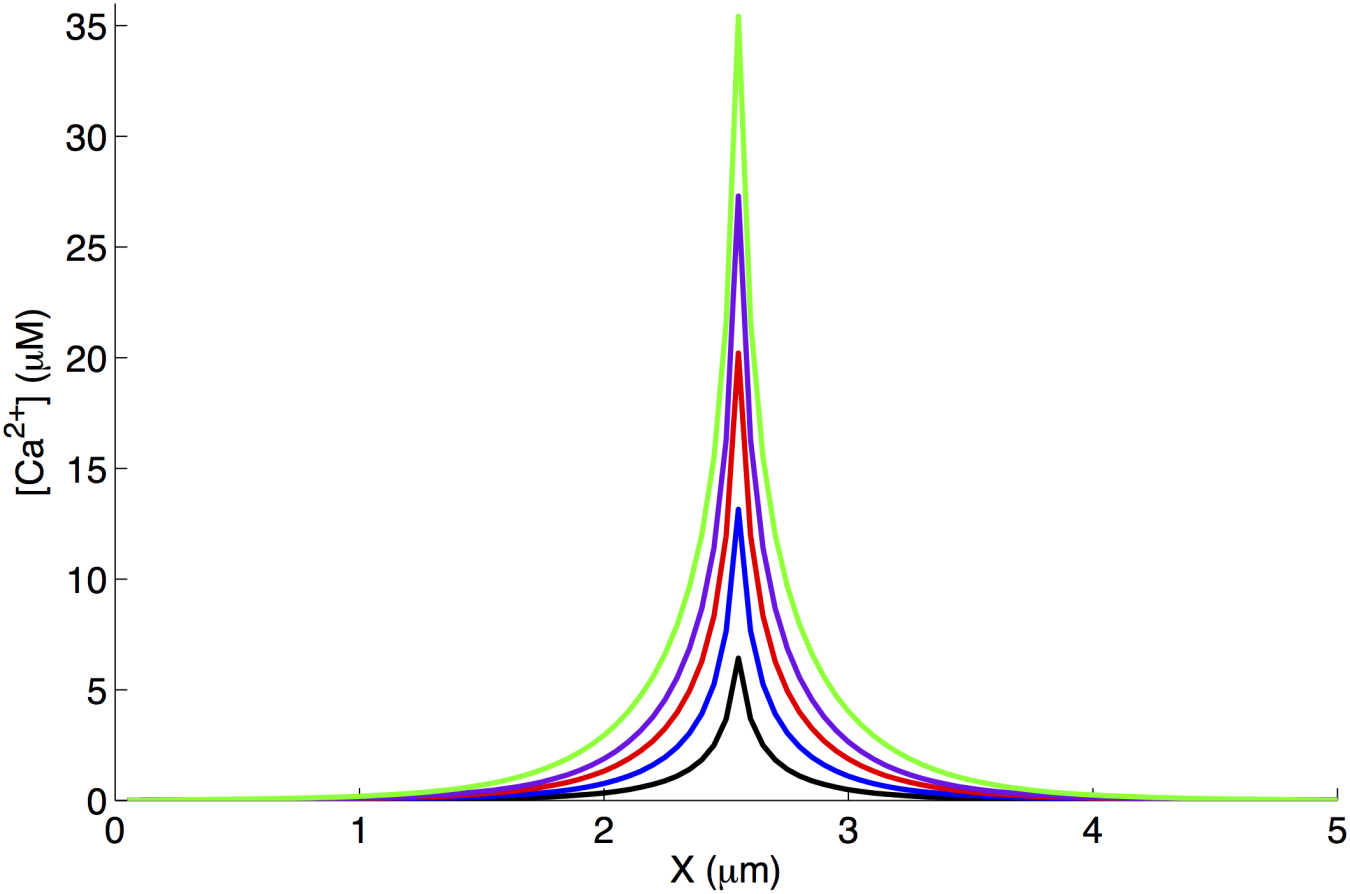
Peak intracellular Ca^2+^ concentration at the pore location during a single opening of type 1 (black), 2 (blue), 3 (red), 4 (purple), and 5 (green) Aβ1–42 pore during the simulation in S3 Fig. Here we show Ca^2+^ concentration along X-axis at the center of Y-axis passing through the pore.

Fig 9 shows a measure of Ca^2+^ influx due to the pore and diffusion in two dimensions modeled as described in S1 Text. We show peak intracellular Ca^2+^ concentration at the pore location due to a single opening of Aβ1–42 pore during the simulation in S3 Fig. We took 2D snapshots of Ca^2+^ influx through different size Aβ1–42 pores during a single opening and show Ca^2+^ concentration along the line passing through the pore at the instant of peak Ca^2+^ at the pore location.

Toxicity level of Aβ pores: To evaluate how Ca^2+^ influx through Aβ pores may disrupt physiological Ca^2+^ signaling pathways in the cell, we investigate the gating of IP_3_R, Ca^2+^-activated chloride (Cl^-^), and BK channels when placed in the vicinity of an Aβ pore. We achieve this by tracking [Ca^2+^] values at various distances from the center of an active Aβ pore (from Fig 9F). Given these [Ca^2+^] values, we then calculate the P_O_ of these three channels versus distance from an open Aβ pore. Its worth mentioning that here we evaluate the P_O_ of these channels at the instant of peak [Ca^2+^] at the Aβ pore. The P_O_ versus distance from the Aβ pore profiles would obviously change with the duration for which the pore is open and its PL and will vary with time as the pore switches from one PL to another, which necessitates the use of Markov chain models developed here for elucidating the disruptions in the gating kinetics of these channels over time.

**Fig 10:**
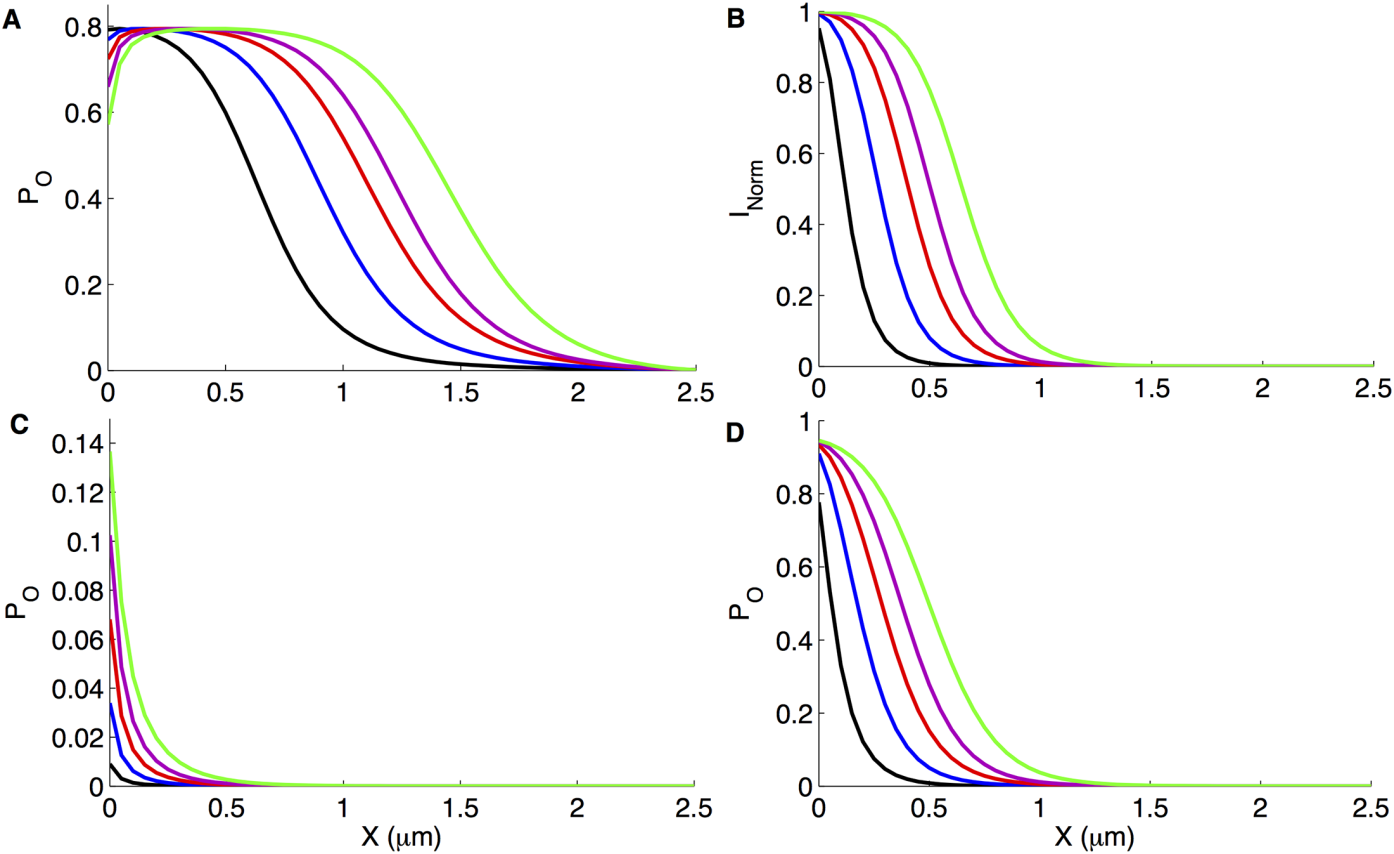
A measure of Aβ pore toxicity in terms of effect on the gating of other channels in the neighborhood. The open probability of IP_3_R at [IP_3_] = 100nM (A), normalized current through Ca^2+^-activated Cl^-^ channel at membrane potential, V = -40mV (B), and open probability of BK channel at V = -60mV (C) and 40mV (D) respectively as a function of distance from the center of an active Aβ pore of type 1 (black), 2 (blue), 3 (red), 4 (purple), and 5 (green).

The [Ca^2+^] values in eq (1) are those given in Fig 9, i.e. Ca^2+^ influx due to an open Aβ pore. The P_O_ of IP_3_R as a function of distance from an open Aβ pore at [IP_3_] = 100nM is shown in Fig 10A. As clear from the figure, the IP_3_R has over 50% probability of opening if within 0.55μm of an active type 1 Aβ pore. While in case of type 5 Aβ pore, this spatial range increases to1.35μm. This is significant considering the fact that IP_3_Rs on the ER can be as close as within 100nm from plasma membrane (and hence Aβ pores). The slight dip in the P_O_ at very small distance is due to the fact that the larger pores bring in enough Ca^2+^ to push IP_3_R to the inhibitory regime. Aβ pores have a similar effect on Ca^2+^-activated chloride (Fig 10B) and BK (Fig 10C and 10D) channels.

To estimate the normalized current through Ca^2+^-activated Cl^-^ channel as a function of Ca^2+^ influx due to an open Aβ pore, we used eq (2) where [Ca^2+^] values are given in Fig 9. Higher conductance Aβ pores clearly have wider spatial range of enhancing the current through Ca^2+^-activated Cl^-^ channels (Fig 10B).

Using the P_O_ of BK channel as a function of [Ca^2+^] due to Aβ pore is given by eq (3). It is interesting to note that, although under physiological conditions BK channel remains mostly closed at resting membrane potential (notice *K*_*D*_ = 35μM at V = -60mV), in the presence of an open Aβ pore it can have more than 13% probability of opening (Fig 10C). The up-regulation of BK channels is even higher at higher membrane potential (Fig 10D). One can expect a similar effect of Aβ pores on other Ca^2+^-regulated channels such as ryanodine receptors.

## Discussion

Due to its significance in memory formation and apoptosis, Ca^2+^ ion is an obvious suspect in AD pathology. Full understanding of Ca^2+^ signaling, particularly its remodeling is crucial for both the etiology of AD and designing efficient therapeutic approaches. Recent observations show that Ca^2+^ permeable Aβ pores are key players in the AD-specific Ca^2+^ signaling machinery [19,20]. As a key step forward in the direction towards the understanding of Ca^2+^ signaling remodeling in AD, here we explore in detail the gating kinetics of Aβ pores with different permeability and open probability.

Depending on the peak permeability level that the Aβ pore can achieve, we put forward five separate models for the gating of the pore. In line with observations [19], the P_O_ and peak permeability and hence the toxicity of Aβ pores increases with time from incubation. The number of pores with SPL_M_ 1 or 2 and low P_O_ decreased while those with SPL_M_ of 3 or higher with higher P_O_ increased. This population behavior hints at the transition of a pore with SPL_M_ 1 and low P_O_ into one with SPL_M_ 2 and higher P_O_, and so on. This behavior is not yet confirmed at the single pore level and requires further investigation.

In the experiments, the transition (at the population level) from pores with the smaller SPL_M_ to those with higher SPL_M_ occurred on the time-scale of several minutes (Fig 1 in [19]). The transition between various states (for example, the transition between a closed state and an open state in the pore with SPL_M_ 1, see Fig 3) on the other hand takes place on the order of milliseconds in most cases. Ideally, there should be one model for the Aβ pore at different stages of its evolution, comprising two time-scales; shorter time-scale for the transitions between states, and a longer time-scale for the transition of a pore with low SPL_M_ to that with higher SPL_M_. In other words, if the five linear chains in Figs 3, 4, 5, 6 and 7 are represented by C_1_, C_2_, ….C_5_, then they should be connected with each other. If the transition from a pore with SPL_M_ 1 to that with SPL_M_ 5 is sequential, then the full model should look like C_1_→C_2_→C_3_→C_4_→C_5_ with a possibility of reversed transitions. Such an extended model would require observing many pores for hundreds of minutes to gather extensive information about the transition from low P_O_ to high P_O_ pore. Furthermore, extensive study is required to understand the behavior of pore both in terms of transition from low P_O_ to high P_O_ pore and transition between different states at a given stage of life under different electrophysiological and chemical conditions. Fortunately, the optical patch-clamp technique is equipped to carryout such future studies that would provide a full understanding of the steps leading to the synthesis of a variety of Aβ pores and gating mechanism [34,35]. Presently, the models developed in this study provide useful insights into the complex gating kinetics of Aβ pore. Furthermore, these models can be used one at a time to study the amount of influx due to Aβ pores with different permeability and open probability (see for example Fig 10).

A close observation of the distributions reveals that the closed-dwell time distribution for the pores having higher permeability shifts significantly to the left as compared to the pores with low permeability. On the other hand, the shift in the lifetime distribution in various open permeability levels is relatively small. This clearly indicates that the higher *P*_*O*_ of the higher conductance pores results largely from the decrease in closed dwell-times (see also Fig 2). It is interesting to note that the observed distributions from permeability levels other than the highest permeability in which various Aβ groups gate have two to three exponential components. Fitting the highest permeability level in all groups on the other hand requires more exponentials and hence more states (more than one or two used in our models). Increasing the number of states in the highest SPL improves the fit to the dwell-time distribution as indicated by the higher *Likelihood* values (not shown). However, since the BIC criteria penalize for higher number of parameters, it gets worst as we add more states to the highest SPL and hence are not warranted.

We found that bigger pores (having high SPL_M_) spend most of their time in the lower permeability levels as compared to the peak permeability level that they can visit. For example, the normalized occupancies of SPL1, 2, 3, 4, and 5 for type 5 pore from both theory and experiment are 0.1311, 0.0116, 0.0043, 0.0025, and 0.00132 respectively. Despite the fact that Ca^2+^ influx through the pore when gating in SPL 5 is five times larger than the flux when gating in SPL 1, the total amount of Ca^2+^ going into the cytoplasm due to SPL 1 would be higher as the pores spend almost 100 times more time in this level as compared to SPL 5. Also, the time that the big pores spend in the low SPLs is much longer as compared to the time spent by small pores (having low SPL_M_) in the low SPLs. For example, the normalized occupancy of SPL1 of type 1 pore from both theory and experiment is 0.00955 (compared to 0.1311 for type 5 pore). From this observation, we infer that the higher permeability pores are more toxic to Ca^2+^ signaling mostly due to the long time that they spend in the low SPLs and to a lesser extent due to their higher flux in higher SPLs.

The topologies of our optimal models reveal that the increase in the pore size does not simply equate to the addition of more states to the existing topology. For example, the transition of type 1 pore to type 2 does not translate to the addition of new states corresponding to SPL 2 to the existing topology for type 1 pore (Fig 3B). The model representing the larger pore rather has a different topology from that representing the smaller pore.

Although time resolutions is the ostensible limitations of optical patch-clamp technique, the 2 ms temporal resolution (500 Hz) achieved in these studies is comparable to these routinely use in lipid bilayer experiments [16, 47], and has allowed us to study other voltage and ligand-gated channels [34,48,49] that, similar to Aβ pores, are known to open stochastically with typical mean open times of few milliseconds [50].

Several observations indicate that the multistep conductance levels did not arise because of co-localization of multiple Aβ pores. In our experiments, we frequently observed apparently synchronous transitions across as many as four amplitude levels and amplitude levels were not distributed as integer multiples as expected if they represent the summated Ca^2+^ flux through spatially unresolved cluster of multiple identical and independent pores Moreover, the spatial resolution of single channel Ca^2+^ fluorescence transients from discrete channels is facilitated because the enormous size (1 mm diameter) of the oocyte results in a low density of channels (< 0.2/μm^2^), even with high expression, and were able to observe stepwise behavior in conditions of much lower overall density of Ab pores. [19].

To conclude, we have developed a set of data-driven models for the kinetics of different types of Aβ pores that have optimal number of parameters and topologies in terms of BIC score. As we show in Fig 10, these models can be used to quantify the effect of Ca^2+^ flux through Aβ pores on cell’s Ca^2+^ homeostasis. Furthermore, this study demonstrates that the massive imaging data obtained from thousands of channels in parallel at the millisecond scale and single channel resolution using TIRF microscopy can be utilized for single molecule (e.g. ion channels) modeling in the same manner as using electrical patch-clamp data. Employing the optical patch-clamp data for Markov chain modeling has the added advantage of being driven by experiments performed under close to physiological conditions.

## Supporting Information

S1 Fig Two-dimensional dwell-time distributions for open-closed intervals of type 1 Aβ42 pore. (A) Two-dimensional distribution obtained from forward and (B) backward analysis of the time-series data. (C) Absolute value of the difference between forward and backward distributions. The color-coded bar represents the square root of the number of events in a given bin (A, B) and the difference between the forward and backward distributions (C) and applies to all panels.
(TIFF)

S2 Fig Time-series traces representing the permeability state of the Aβ1–42 pore as a function of time. Sample traces from type 1 (A), type 2 (B), type 3 (C), type 4 (D), and type 5 (E) pores. Black and red lines represent the experimental data and the traces given by the best models respectively.
(TIFF)

S3 Fig Traces representing the changes in the intracellular Ca^2+^ concentration due to Aβ1–42 pores as a function of time. The left and right columns respectively show sample fluorescence traces from experiments and Ca^2+^ concentration traces from the best model for type 1 (A), type 2 (B), type 3 (C), type 4 (D), and type 5 (E) pores.
(TIFF)

S1 Table Rate constants for the One SPL Aβ1–42 pore models. (DOCX)

S2 Table Rate constants for the two SPLs Aβ1–42 pore models. (DOCX)

S3 Table Rate constants for the three SPLs Aβ1–42 pore models. (DOCX)

S4 Table Rate constants for the four SPLs Aβ1–42 pore models. (DOCX)

S5 Table Rate constants for the five SPLs Aβ1–42 pore models. (DOCX)

S1 Text Supplementary Information. (PDF)
