# Supplementary figures and images for: Analyzing and Modeling the Kinetics of Amyloid Beta Pores Associated with Alzheimer’s Disease Pathology
Source: PLoS One. 2015 Sep 8;10(9):e0137357. doi: 10.1371/journal.pone.0137357 (PMC4562663; doi:10.1371/journal.pone.0137357)

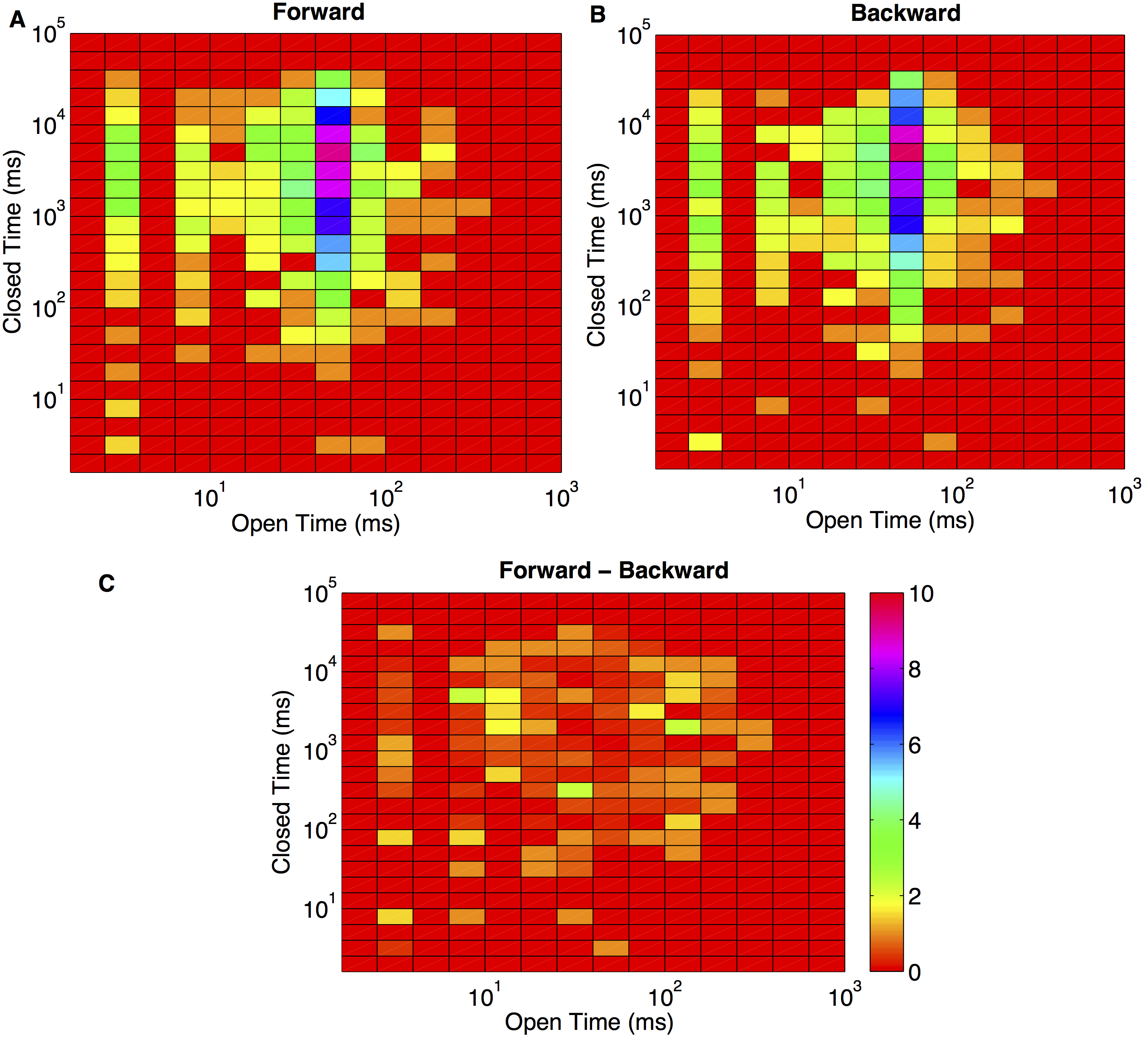

Supplement: S1 Fig — (A) Two-dimensional distribution obtained from forward and (B) backward analysis of the time-series data. (C) Absolute value of the difference between forward and backward distributions. The color-coded bar represents the square root of the number of events in a given bin (A, B) and the difference between the forward and backward distributions (C) and applies to all panels. (TIFF) [file pone.0137357.s001.tiff]

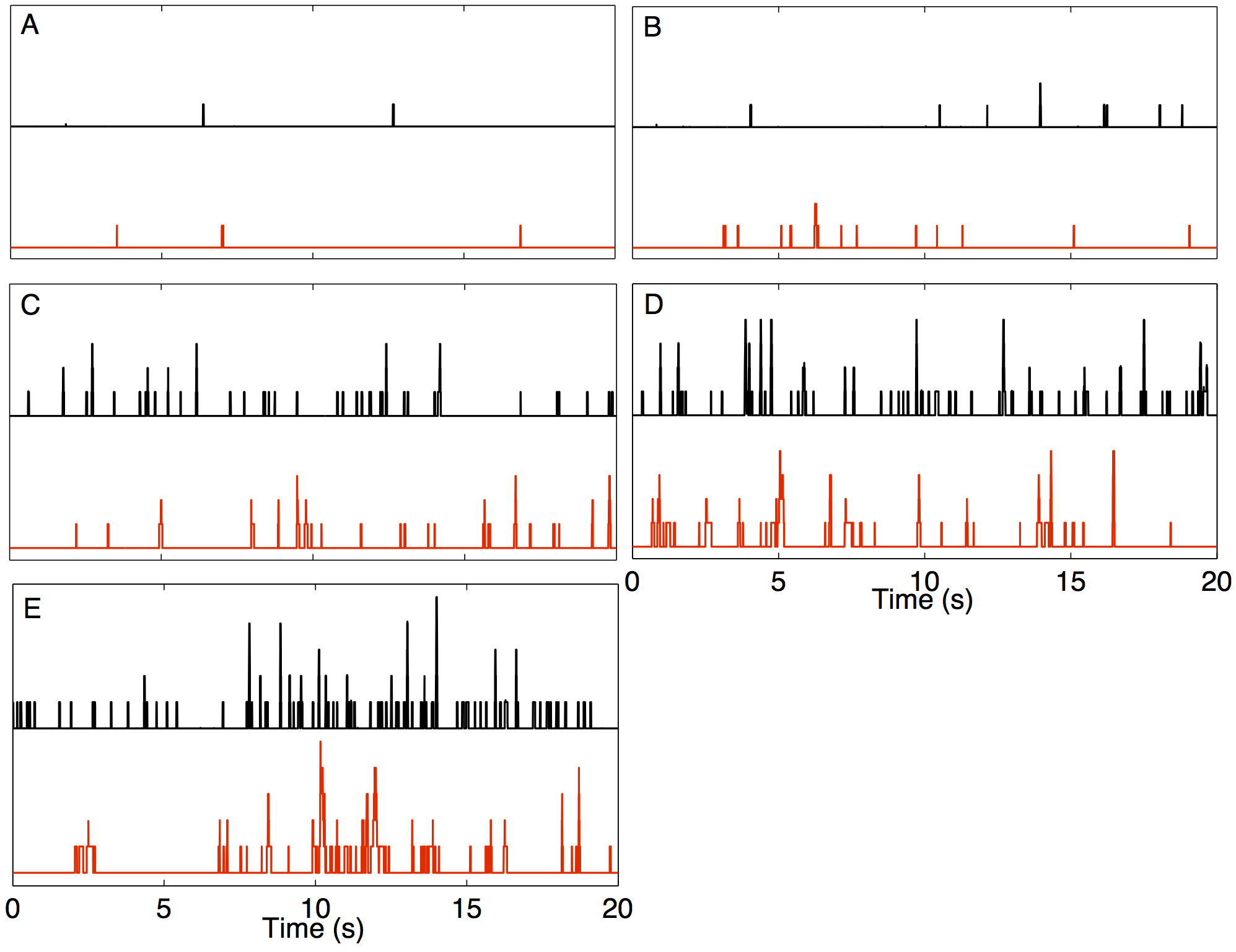

Supplement: S2 Fig — Sample traces from type 1 (A), type 2 (B), type 3 (C), type 4 (D), and type 5 (E) pores. Black and red lines represent the experimental data and the traces given by the best models respectively. (TIFF) [file pone.0137357.s002.tiff]

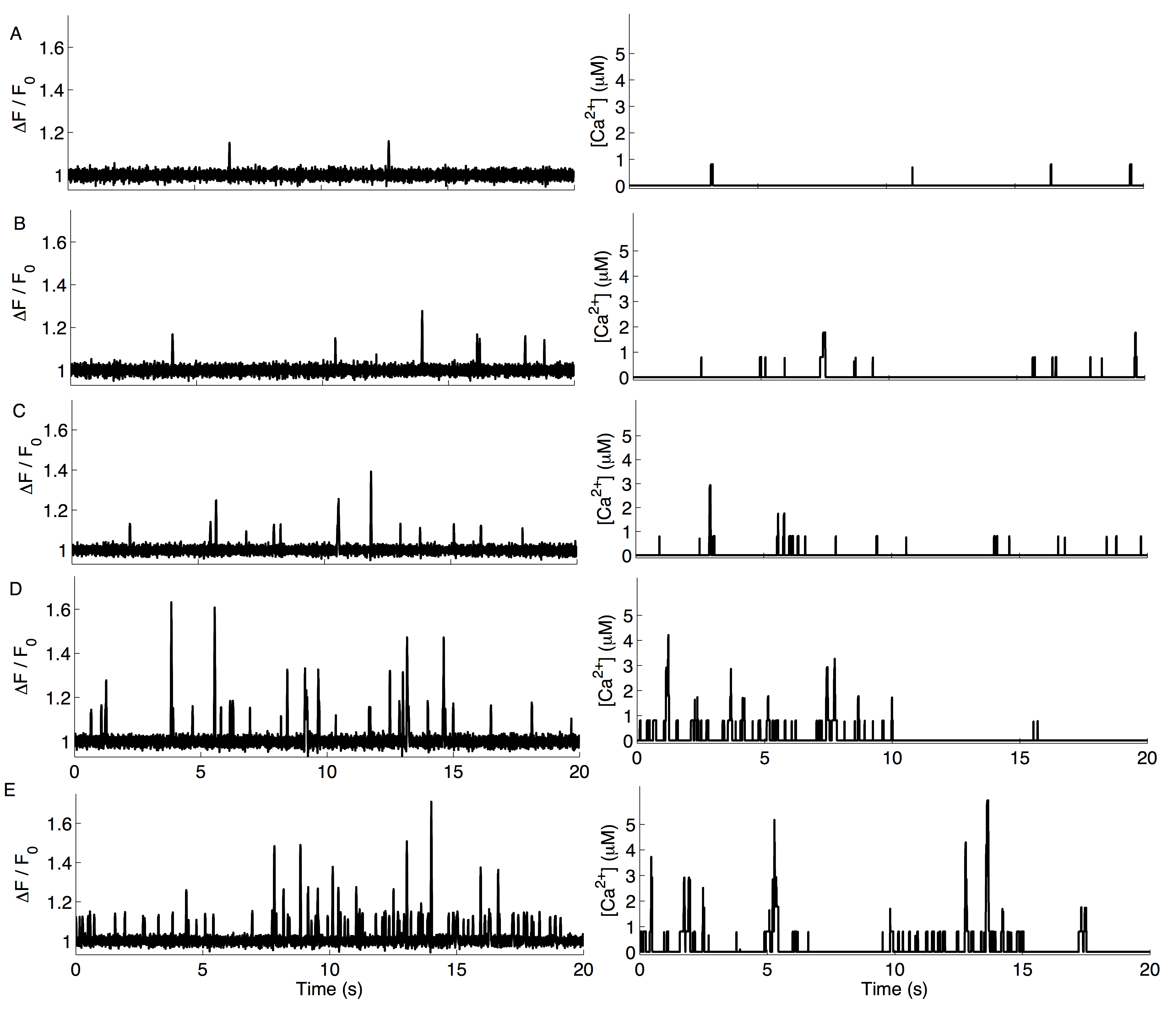

Supplement: S3 Fig — The left and right columns respectively show sample fluorescence traces from experiments and Ca2+ concentration traces from the best model for type 1 (A), type 2 (B), type 3 (C), type 4 (D), and type 5 (E) pores. (TIFF) [file pone.0137357.s003.tiff]
