# Supplementary material for: Analyzing and Modeling the Kinetics of Amyloid Beta Pores Associated with Alzheimer’s Disease Pathology
Source: PLoS One. 2015 Sep 8;10(9):e0137357. doi: 10.1371/journal.pone.0137357 (PMC4562663; doi:10.1371/journal.pone.0137357)
Supplement: S1 Table — (DOCX) [file pone.0137357.s004.docx]

**S1 Table**

| Initial State Final State Rate (sec^-1^) | Initial State Final State Rate (sec^-1^) |
| --- | --- |
| Simplest Model | |
| 0 1 0.34361 | 1 0 35.606 |
| Best Model | |
| 0a 1a 0.2260075  1a 0a 35.329858  0a 1b 0.0164894  1b 0a 3.4987589 | 0b 1b 0.7331798  1b 0b 18.184035  0c 1b 1.5803847  1b 0c 15.315078 |
