# Supplementary material for: Analyzing and Modeling the Kinetics of Amyloid Beta Pores Associated with Alzheimer’s Disease Pathology
Source: PLoS One. 2015 Sep 8;10(9):e0137357. doi: 10.1371/journal.pone.0137357 (PMC4562663; doi:10.1371/journal.pone.0137357)
Supplement: S2 Table — (DOCX) [file pone.0137357.s005.docx]

**S2 Table**

| Initial State Final State Rate (sec^-1^) | Initial State Final State Rate (sec^-1^) |
| --- | --- |
| Simplest Model | |
| 0 1 1.0678  1 0 35.487 | 1 2 5.4421  2 1 50.959 |
| Best Model | |
| 0a 1a 0.5354  1a 0a 49.8240  0b 1b 2.36028  1b 0b 30.63257  1a 1b 264454.6009  1b 1a 89602.893 | 1a 2a 19.9427  2a 1a 47.372  1b 2b 0.54356  2b 1b 2727.114  2a 2b 100.4004  2b 2a 71849.153 |
