# Supplementary material for: Analyzing and Modeling the Kinetics of Amyloid Beta Pores Associated with Alzheimer’s Disease Pathology
Source: PLoS One. 2015 Sep 8;10(9):e0137357. doi: 10.1371/journal.pone.0137357 (PMC4562663; doi:10.1371/journal.pone.0137357)
Supplement: S3 Table — (DOCX) [file pone.0137357.s006.docx]

**S3 Table**

| Initial State Final State Rate (sec^-1^) | Initial State Final State Rate (sec^-1^) |
| --- | --- |
| Simplest Model | |
| 0 1 1.7718  1 0 32.455  1 2 5.9403 | 2 1 64.541  2 3 13.189  3 2 52.442 |
| Best Model | |
| 0a 1a 3.94611  1a 0a 30.54968  0b 1b 0.8161  1b 0b 35.3143  1a 1b 7.13335  1b 1a 3.10408  1a 2a 1.119478  2a 1a 3.10408  1a 2b 4.059075  2b 1a 35.318798 | 1b 2b 6.241899  2b 1b 23.63388  2a 2b 36431.8019  2b 2a 6105.4766  2a 3a 1.20408  3a 2a 1639.846  2b 3b 22.88655  3b 2b 53.949  3a 3b 20851.429  3b 3a 6.048 |
