# Supplementary material for: Analyzing and Modeling the Kinetics of Amyloid Beta Pores Associated with Alzheimer’s Disease Pathology
Source: PLoS One. 2015 Sep 8;10(9):e0137357. doi: 10.1371/journal.pone.0137357 (PMC4562663; doi:10.1371/journal.pone.0137357)
Supplement: S4 Table — (DOCX) [file pone.0137357.s007.docx]

**S4 Table**

| Initial State Final State Rate (sec^-1^) | Initial State Final State Rate (sec^-1^) |
| --- | --- |
| Simplest Model | |
| 0 1 2.0975  1 0 24.317  1 2 6.7412  2 1 64.289 | 2 3 26.139  3 2 72.008  3 4 42.205  4 3 66.029 |
| Best Model | |
| 0b 1b 0.742337  1b 0b 21.3346  0a 1a 5.36303  1a 0a 20.68043  0a 1b 1.33696  1b 0a 11.49888  1b 2b 9.05892  2b 1b 179.242 | 1a 2a 5.93618  2a 1a 46.5023  2b 2a 868.78755  2a 2b 154.2163  2a 3 31.08345  3 2a 71.6531  3 4 40.60456  4 3 64.73825 |
