# Supplementary material for: Analyzing and Modeling the Kinetics of Amyloid Beta Pores Associated with Alzheimer’s Disease Pathology
Source: PLoS One. 2015 Sep 8;10(9):e0137357. doi: 10.1371/journal.pone.0137357 (PMC4562663; doi:10.1371/journal.pone.0137357)
Supplement: S5 Table — (DOCX) [file pone.0137357.s008.docx]

**S5 Table**

| Initial State Final State Rate (sec^-1^) | Initial State Final State Rate (sec^-1^) |
| --- | --- |
| Simplest Model | |
| 0 1 3.1285  1 0 20.565  1 2 5.6032  2 1 62.976  2 3 29.287 | 3 2 77.886  3 4 42.211  4 3 73.603  4 5 32.276  5 4 66.83 |
| Best Model | |
| 0a 0b 1.01823  0b 0a 0.762016  0a 1a 7.3944  1a 0a 24.202976  1a 1b 1.09168  1b 1a 6.5740  2a 1a 62.77213  1a 2a 6.49818  2a 2b 12.0142 | 2b 2a 9808542.88  2a 3a 29.563197  3a 2a 80.2461  2b 3b 1.104767  3b 2b 10.9655  3a 4 43.8887  4 3a 80.27613  4 5 38.6061  5 4 71.21032 |
