# Supplementary material for: Analyzing and Modeling the Kinetics of Amyloid Beta Pores Associated with Alzheimer’s Disease Pathology
Source: PLoS One. 2015 Sep 8;10(9):e0137357. doi: 10.1371/journal.pone.0137357 (PMC4562663; doi:10.1371/journal.pone.0137357)
Supplement: S1 Text — (PDF) [file pone.0137357.s009.pdf]

Supporting information:
Analyzing and modeling the kinetics of amyloid beta pores associated with Alzheimer's disease pathology

Ghanim Ullah[1], Angelo Demuro[2], Ian Parker[2], and John E Pearson[3]

[1]Department of Physics, University of South Florida, Tampa, FL 33620, USA
[2]Department of Neurobiology and Behavior, University of California, Irvine, CA 92697, USA
[3]Theoretical Biology and Biophysics, Los Alamos National Laboratory, Los Alamos, NM 87545, USA

Supplementary methods

Checking for detailed balance: To show that the gating kinetics of Aβ42 pores is consistent with the thermodynamic principle of detailed balance, we followed the procedure outlined in [1] (see also [2]). We obtained two-dimensional dwell time distributions of adjacent open and closed times in the forward direction from the time-series data obtained from type 1 (pores that can open up to a maximum permeability of SPL 1) Aβ42 pores. We used the logs of the open duration and the following closed duration to locate a bin on the x-y plane with 5 bins per log unit. The same procedure was repeated by logarithmically binning the pairs of adjacent open and closed intervals in the backward (reverse) direction on the time-series data. A reversible gating mechanism gives similar results for forward and backward analysis of the data. Forward and backward two-dimensional distributions based on 217 segments (each 20 s long) are shown in S1 Fig. As in [1], we used the χ^2 test for the significant differences between the two distributions. A χ^2 value is calculated as

$$\chi^2 = \sum_{i,j} \frac{[F(i,j) - E(i,j)]^2}{E(i,j)} + \frac{[B(i,j) - E(i,j)]^2}{E(i,j)} \qquad (1)$$

Where *F(i,j)* and *B(i,j)* refer to the number of events in bin *(i,j)* for the forward and backward distributions, respectively, and $E(i,j) = [F(i,j)+B(i,j)]/2$ is the average number of events in bin *(i,j)* for both the forward and backward distributions. The summation in equation (1) is restricted to those bins with five or more events in both the forward and backward distributions, and the degrees of freedom, *D*, is given by the total number of such pairs [1]. Finally, we calculated the approximate normal deviate $Z = \sqrt{2\chi^2} + \sqrt{2D-1}$ to estimate the significance of the difference. Observed differences are significant at the 5% level if $Z > 1.96$ (Snedecor and Cochran, 1989). Our analysis gave $Z = -1.5518$, indicating that our data are consistent with the microscopic reversibility hypothesis. We reached the same conclusion for other types of pores.

Stochastic scheme of the pore gating: To determine the state in which the Aβ42 pore is gating at a given time, we employed the procedure outlined in [2,3]. Briefly, we considered the best Markov chain model developed in the main text for the pore type under consideration. For example, if the pore under consideration is type 1, the model in Fig. 3B (main text) is used for its

gating. Next, we determined the transition probabilities for the pore at a given time. For example, if the pore is in state **0a**, possible transitions are to states **1a** and **1b** (Fig. 3B of main text). For a sufficiently small time interval Δt, the probabilities for these two transitions are given by $P_{0a1a} = K_{0a1a}\,\Delta t$ and $P_{0a1b} = K_{0a1b}\,\Delta t$ respectively. The probability for the pore to remain in state **0a** is $P_{0a0a} = 1 - P_{0a1a} - P_{0a1b}$. The unit interval was divided into three subintervals of length P_{0ax}, where x represents the three states to which the pore can make transition (including the current state). If a random number drawn from a uniform distribution over the interval Δt falls into the subinterval P_{0ax}, the corresponding transition is performed. The time interval was kept small enough for the linear dependence of P_{xx} on the time interval to remain valid.

Simulation of Ca^{2+} concentration traces: To generate time-series traces representing the changes in Ca^{2+} concentration ($[Ca^{2+}]$) due to Aβ42 pore, we considered a single pore at the center of a 25μm^2 square patch of plasma membrane. Calcium concentration on the cytoplasm side of the pore is controlled by diffusion, the flux coming in from the extracellular space through the pore, *J*, and the concentration of free dye buffer, $[b_{dye}]$. Thus the rate equations for $[Ca^{2+}]$ and $[b_{dye}]$ are given as

$$\frac{\partial [Ca^{2+}]}{\partial t} = D_{Ca}\nabla^2[Ca^{2+}] + J\delta(x_{pore}, y_{pore}) + k^r([B_{dye}] - [b_{dye}]) - k^f[Ca^{2+}][b_{dye}] \quad (2)$$

$$\frac{\partial [b_{dye}]}{\partial t} = D_{dye}\nabla^2[b_{dye}] + k^r([B_{dye}] - [b_{dye}]) - k^f[Ca^{2+}][b_{dye}] \quad (3)$$

Where D_{Ca} = 223μm^2/sec , ∇^2, δ, (x_{pore}, y_{pore}) =(0,0), k^f = 100/μMsec, k^r = 25/sec, and D_{dye} = 200μm^2/sec is the diffusion coefficient of Ca^{2+}, Laplace operator in rectangular coordinates, Dirac delta function, (x, y) coordinates of the pore, rate of Ca^{2+} binding and unbinding to the dye, and diffusion coefficient of dye. All these parameters are based on experimental observations (see [4] for references). Following the experimental preparation [5], a total dye concentration, $[B_{dye}]$ = 40μM, was used in the cytoplasm. Ca^{2+} flux through the pore is given by the following equation

$$J = \frac{I}{2 \times F \times \delta V} \quad (4)$$

Where *I*, *F*, and δV is the current through the pore, Faraday's constant, and volume of the hemisphere over the pore having a radius of r_{pore}. Demuro et al., [5] estimated a current of 0.05 – 0.5pA through Aβ pores. We used *I* = 0.05pA per permeability level. That is, the pore allows a current of 0.05pA when gating in SPL 1, 0.1pA when in SPL 2 and so on. r_{pore} = 1nm is based on the value obtained from AFM study of Aβ1-42 pore [6] (see also [7-9]). A spatial grid size of 0.05μm and time step of 1μsec was used for solving the diffusion equations.

Supplementary results

Time-series traces: Twenty seconds long sample traces representing the permeability level in which the pore is gating as a function of time for the five groups of A *β* 42 pores are shown in S2 Fig. The black line in each panel represents the observed trace while the red line representing the trace given by the best model for that group of pores is shown for comparison. The traces given by the models closely resemble the observed traces. For clarity, all traces are scaled according to those obtained from the pore with a maximum permeability of SPL 5.

In S3 Fig., we compare traces representing Ca^{2+} influx through A β 1-42 pore as a function of time from experiment and models. The right column displays the observed fluorescence changes (Δ F) with respect to the base level fluorescence (F_0) representing relative changes in average Ca^{2+} concentration due to the opening and closing of the pore. Average intracellular Ca^{2+} concentrations from the point of interest (1 μ m^2 centered on the pore), given by the models are shown in the right column.

Over-parameterization test: To avoid over-parameterized models, we performed Kienker transformations [10] in order to search for Bauer-Kienker uncoupled (BKU) canonical forms of the models developed above. In the BKU canonical form first described by Baure et al. [11] and and Kienker [10], only the transitions between states having different conductances are allowed with no links between states having the same conductance. The detail of Kienker transformations is given in [10] and is summarized as follows.

Finite state Markov chains obey an evolution equation $dP/dt=PQ,$ where P is a vector with $P_i\ (t)$ being the probability of the system in state i at time t and Q is the generator matrix for the Markov chain with elements Q_{ij} the transition rate from state i to j and $Q_{ii} = -\sum_{j\neq i} Q_{ij}$ [12-16].

In case of one closed and N open aggregates, Q can be partitioned as

$$\begin{pmatrix} Q_{O1O1} & \cdots & Q_{O1ON} & Q_{O1C} \\ \vdots & \cdots & \vdots & \vdots \\ Q_{ONO1} & \cdots & Q_{ONON} & Q_{ONC} \\ Q_{CO1} & \cdots & Q_{CON} & Q_{CC} \end{pmatrix} \qquad (5)$$

Where the elements of sub-matrix Q_{XY} contain all transition rates from aggregate X to aggregate Y. Kienker [10] proved that two models with generator matrices Q and $\tilde{Q}$ are equivalent if and only if they can be related by similarity transformation $\tilde{Q} = S^{-1}QS$, where S is of the form

$$\begin{pmatrix} S_{O1O1} & \cdots & 0 & 0 \\ \vdots & \cdots & \vdots & \vdots \\ 0 & \cdots & S_{ONON} & 0 \\ 0 & \cdots & 0 & S_{CC} \end{pmatrix} \qquad (6)$$

Where $S_{XX}\, u_X = u_X$ and u_X is a column vector of ones with dimension equal to the number of states in aggregate X. The sub-matrices of $\tilde{Q}$ are given as $\tilde{Q}_{XY} = S_{XX}^{-1} Q_{XY} S_{YY}$.

References

1. Song L. and Magleby K. (1994) Testing for microscopic reversibility in the gating of maxi k+ channels using two-dimensional dwell-time distributions. Biophys. J., 67:91–104.

2. Ullah G., Mak D. O. D., and Pearson J. E. (2012). A data-driven model of a modal gated ion channel: The inositol 1, 4, 5-trisphosphate receptor in insect Sf9 cells. J. Gen. Physiol. 140:159-173.

3. Ullah G. and Jung P. (2006). Modeling the statistics of elementary calcium release events. Biophys. J., 90:3485-3495.

4. Ullah G., Parker I., Mak D. O. D., and Pearson J. E. (2012a). Multi-scale data-driven modeling and observation of calcium puffs. Cell Calcium, 52:152-160.

5. Demuro A., Smith M., and Parker I. (2011) Single-channel Ca^{2+} imaging implicates Abeta1-42 amyloid pores in Alzheimer's disease pathology. J. Cell. Biol., 195:515–524.

6. Lin H., Bhatia R., and Lal R. (2001) Amyloid beta protein forms ion channels: implications for Alzheimer's disease pathophysiology. FASEB J., 15:2433-2444.

7. Shafrir Y., Durell S., Arispe N., and Guy H. R. (2010) Models of membrane - bound Alzheimer's Abeta peptide assemblies. Proteins: Structure, Function, and Bioinformatics, 78(16), 3473-3487.

8. Connelly, L., Jang, H., Teran Arce, F., Ramachandran, S., Kagan, B. L., Nussinov, R., and Lal, R. (2012) Effects of point substitutions on the structure of toxic Alzheimer's β-amyloid channels: atomic force microscopy and molecular dynamics simulations. Biochemistry, 51(14), 3031-3038.

9. Connelly L., Jang H., Teran Arce F., Capone R., Kotler S. A., Ramachandran S., Kagan B. L., Nussionov R., and Lal R. (2012) Atomic force microscopy and MD simulations reveal pore-like structures of all-D-enantiomer of Alzheimer's β-amyloid peptide: relevance to the ion channel mechanism of AD pathology. The Journal of Physical Chemistry B, 116(5), 1728-1735.

10. Kienker P. (1989) Equivalence of aggregated Markov models of ion-channel gating. Proc. R. Soc. London B. Biol. Sci., 236: 269-309.

11. Bauer R. J. and Bowman B. F. and Kenyon J. L. (1987) Theory of the kinetic analysis of patch-clamp data. Biophys. J., 52: 961-978.

12. Colquhoun D. and Hawkes A. G. (1981) On the stochastic properties of single ion channels. *Proc. R. Soc. London B,* 211:205-236.

13. Colquhoun D. and Hawkes A. G. (1982) On the stochastic properties of bursts of single ion channel openings and of clusters of bursts. *Philos. Trans. R. Soc. London B,* 300:1-59.

14. Fredkin, D. R., Montal, M., and Rice, J. A. (1985) *Proceedings of the Berkeley Conference in Honor of Jerzy Neyman and Jack Kiefer*, eds. Le Cam, L. M. & Olshen, R. A. (Wadsworth, Belmont, CA), pp. 269-289.

15. Fredkin, D. R. and Rice, J. A. (1986) On Aggregated Markov Processes. *J. Appl. Probability,* 23: 208-214.

16. Bruno W. J., Yang, J., and Pearson J. E. (2005). Using independent open-to-closed transitions to simplify aggregated Markov models of ion channel gating kinetics. Proc. Natl. Acad. Sci. USA. 102:6326-6331.
